# Psychological factors associated with foot and ankle pain: a mixed methods systematic review

**DOI:** 10.1186/s13047-021-00506-3

**Published:** 2022-02-03

**Authors:** Matthew Cotchett, Nicoletta Frescos, Glen A. Whittaker, Daniel R. Bonanno

**Affiliations:** 1 Discipline of Podiatry, School of Allied Health, Human Services and Sport, La Trobe University, Melbourne, Victoria 3086 Australia; 2 La Trobe Sport and Exercise Medicine Research Centre, La Trobe University, Melbourne, Victoria 3086 Australia

**Keywords:** Mental health, Musculoskeletal pain, Plantar fasciitis, Achilles tendon

## Abstract

**Background:**

Foot and ankle pain is common, and generally viewed through a biomedical lens rather than applying a biopsychosocial model. The objectives of this review were to evaluate: (1) the psychosocial characteristics of participants with foot/ankle pain compared to participants without foot/ankle pain; (2) the association between psychosocial factors with pain and function in people with foot/ankle pain; and (3) understand the psychosocial factors associated with the lived experience of foot/ankle pain.

**Methods:**

A mixed methods systematic review was conducted according to the PRISMA guidelines and guided by the Joanna Briggs Institute methodology for mixed methods systematic reviews. The databases MEDLINE, Embase, CINAHL, SPORTDiscus, PsychInfo, and Scopus were searched. The Mixed Methods Assessment Tool was used to evaluate study quality. A convergent segregated approach was used to synthesise and integrate quantitative and qualitative data.

**Results:**

Eighteen studies were included, consisting of 13 quantitative, 4 qualitative and 1 mixed methods study. The overall quality of the studies was considered high. Integration of the quantitative and qualitative data were not possible due to the disparate nature of the included studies. A narrative synthesis of the quantitative data revealed that negative emotional and cognitive factors were more common in people with foot/ankle pain compared to those without foot/ankle pain. A significant association was also found between emotional distress with foot pain and foot function in some people with plantar heel pain. In addition, kinesiophobia and pain catastrophising were significantly associated with impaired foot function, and pain catastrophising was significantly associated with first step pain in people with plantar heel pain. The qualitative data revealed emotional impacts, physical challenges, and a loss of self which was individual and unpredictable.

**Conclusions:**

This review provides evidence that negative psychological constructs are greater in participants with foot/ankle pain compared to those without foot/ankle pain, although the cross-sectional nature of the study designs included in this review reduces the certainty of the evidence. These findings indicate that psychological constructs are associated with foot/ankle pain. Further research should evaluate the predictive ability of multidimensional screening tools to identify patients at risk of developing persistent foot/ankle pain.

**Supplementary Information:**

The online version contains supplementary material available at 10.1186/s13047-021-00506-3.

## Introduction

Foot and ankle (foot/ankle) pain is common in the community. Foot pain is estimated to have a prevalence between 13 and 36% [1] and ankle pain has an approximate prevalence of 12% [2]. Compared to other body regions, the foot/ankle is the third most common site of self-reported joint pain for adults aged over 55 years [3]. The presence of foot/ankle pain is a risk factor for functional impairments such as locomotor disability [4], impaired balance [5], and increased risk of falls [6]. In addition to functional impairments, the presence of foot/ankle pain is a risk factor for reduced health-related quality of life [7].

Factors associated with the presence of foot/ankle pain should be viewed through a biopsychosocial model, which describes foot/ankle pain as a result of the interaction between biological, psychological and social factors [8]. This model shifts the focus from the pathophysiological processes associated with nociception and reinforces the influence of a person’s emotional state, cognitive processes, and subsequent behaviour on pain [9]. Interestingly, there is no evidence for any musculoskeletal condition that a direct relationship exists between tissue pathology and/or a radiographic finding, and a person’s experience of pain, including its intensity [10].

Emotional factors such as depression, anxiety and general indicators of emotional distress are more common in people with persistent pain than in pain-free controls for a range of conditions (e.g., mixed, back, head, neck, fibromyalgia, arthritis) [11]. Cognitive factors, which relate to how individuals think about their pain, have also been found to be associated with the experience of pain. For example, pain catastrophising is associated with increased pain intensity, pain-related disability, and psychological distress - even when controlling for level of physical impairment [12]. The perception of pain may also be associated with coping behaviours that produce positive outcomes, or behaviours that are maladaptive such as avoidance behaviours, which might be associated with further dysfunction, depression and pain [13].

An awareness of psychological factors is important for health professionals to broadly understand an individual’s experience of pain. If negative psychological factors are found to be more common, or more severe, in individuals with foot/ankle pain or associated with their pain or function, health professionals can assess and address these factors. A recent systematic review evaluated the association between psychological factors and clinical outcomes in tendinopathy, with studies related to plantar heel pain (*n* = 3) and Achilles tendinopathy (*n* = 1) being among the included studies. The review concluded a weak to moderate strength of association between psychological factors and pain, disability and physical functional outcome in tendinopathy from 10 observational studies. Longitudinal data failed to show a predictive relationship between baseline psychological factors and long-term outcomes [14]. Although these findings are of interest, the review needs to be considered in light of some limitations. For example, the review did not include other musculoskeletal conditions of the foot/ankle (e.g., osteoarthritis) or qualitative research related to the lived experience of people with foot/ankle pain. The synthesis and integration of qualitative data applied to research questions can add value by providing more insightful ways to understand the nature of an individual’s experience, and in this case the biopsychosocial aspects of foot/ankle pain.

Therefore, the aim of this mixed methods review is to synthesise quantitative and qualitative data to investigate psychosocial factors in people with foot/ankle pain. This will be achieved by (i) determining the psychosocial characteristics of participants with foot/ankle pain compared to participants without foot/ankle pain, (ii) determining psychosocial factors that are associated with pain and function in people with foot/ankle pain, and (iii) identifying psychosocial factors associated with a participant’s lived experience of foot/ankle pain.

## Methods

The protocol was registered with PROSPERO (CRD42020200764) and no deviations to the protocol were made after registration. The review is reported according to the Preferred Reporting Items for Systematic Reviews and Meta-Analyses (PRISMA) guidelines [15] and guided by the Joanna Briggs Institute (JBI) methodology for mixed methods systematic reviews [16].

### Inclusion criteria

#### Population

Adult participants with foot/ankle pain of at least 1 month duration were included. We excluded studies in which participants experienced pain associated with surgery, fractures, infections, tumours, systemic inflammatory disorders, neurological disorders, and participants that had concurrent pain in regions outside the foot/ankle.

#### Exposure

The quantitative component of this review considered studies that evaluated the likelihood of having foot/ankle pain in the presence of a psychological factor(s) and also studies that evaluated the association between psychological factors with foot pain and foot function.

#### Phenomena of interest

Qualitative studies that investigated the lived experience of people with foot/ankle pain were considered for analysis.

#### Outcomes

The quantitative component of this review considered studies which reported at least one self-reported outcome that evaluated a psychological variable.

#### Context

The qualitative component of the review included studies that investigated the lived experience of community dwelling people with foot/ankle pain.

### Types of studies

This review considered quantitative, qualitative and mixed methods studies. Quantitative studies included randomised controlled trials, cohort, cross-sectional and case-control designs. Qualitative studies included data relevant to the proposed phenomena of foot/ankle pain. Methodologies of interest included qualitative design, phenomenology, ethnography, grounded theory, action research and the qualitative component of mixed methods studies. Mixed methods studies were only included if the data from the quantitative and qualitative components of the study could be extracted and synthesised. Narrative reviews, editorials, conference abstracts, non-published studies (e.g., theses), single case studies, and non-primary literature (e.g., systematic reviews) were excluded.

### Search strategy

The search strategy was developed and refined through discussion with the authors and a librarian. The search strategy, including all identified keywords and index terms were adapted for each included database. In March 2021, the following electronic databases were searched: MEDLINE, Embase, CINAHL, SPORTDiscus, PsychInfo, and Scopus. A full electronic search strategy from the CINAHL database is included in Supplementary file 1. The reference lists of all included articles were hand searched for studies meeting the inclusion criteria.

### Study selection

Following the search, all identified citations were collated and uploaded into EndNote X9 (Clarivate Analytics, PA, USA) and duplicates were removed. Relevant titles and abstracts were independently screened by pairs of reviewers (MC and NF) with disagreements resolved by a third team member (DRB or GAW). Full-text articles were retrieved and assessed against the inclusion criteria by two independent reviewers (MC and NF). Full-text studies that did not meet the inclusion criteria were excluded, and reasons for exclusion were provided. Disagreements that arose between the reviewers were resolved through discussion with a third reviewer (DRB or GAW).

### Assessment of methodological quality

Two reviewers (DRB and GAW) independently assessed the methodological quality of the included studies using the Mixed Methods Appraisal Tool (MMAT) [17]. The MMAT is a reliable [18] and valid [19] quality assessment tool designed to facilitate critical appraisal of studies included in systematic mixed studies reviews (i.e., reviews that include qualitative, quantitative, and mixed methods studies) [17]. All included studies were initially assessed against two screening questions relating to the research question/s and the studies’ data to ensure they were empirical studies. Following this, the quality of each study was appraised by rating the criteria in the relevant category based on the study design. All methodological quality criteria were documented as ‘yes’, ‘no’ or ‘can’t tell’, with the latter recorded when insufficient information was available to provide a ‘yes’ or ‘no’ response. Reviewers met and discussed any discrepancies until all ratings were agreed.

### Data extraction

For quantitative studies, four reviewers (MC, DRB, GAW and NF) extracted data including specific details about the study design and setting, and participant characteristics such as age, gender, BMI, level of education, degree of pain, level of function, psychosocial outcome measure and outcomes of significance to the review question. The lead author (MC) checked all extracted data for accuracy.

For the qualitative component, two reviewers (MC and NF) independently extracted study information using the *JBI QARI Data Extraction Tool for Qualitative Research* [16] and disagreements were resolved through discussion. Through repeated reading of the included studies, extracted *findings* were identified as a verbatim extract of a theme or metaphor that was developed through the authors’ data analysis. Each *finding* that was extracted was accompanied by an *illustration* that informed the finding and included a direct quotation of a participant’s voice. The two independent reviewers then allocated a level of credibility to each extracted finding, as follows:

- Unequivocal – findings with illustrations that are beyond reasonable doubt and thus not open to challenge.
- Credible – findings with an illustration that are not closely associated with the finding and is open to challenge.
- Unsupported – findings not supported by the data.

If more than one illustration was provided for a finding, the illustration with the highest level of credibility was allocated to the finding. Unsupported findings, where there was no quotation of the participant voice, were excluded from further analysis and synthesis.

### Data synthesis

A narrative synthesis of quantitative data was used to evaluate psychological characteristics in people with foot/ankle pain compared to people without foot/ankle pain and also to investigate the association between psychological factors and measures of pain and function. Statistical pooling was not possible.

Meta-aggregation [20] was used to synthesise qualitative data to understand the lived experience of participants with foot/ankle pain including the psychosocial factors associated with their experience. To complete this synthesis, unequivocal or credible findings were independently grouped into categories by MC and NF (Supplementary file 2) based on similarity of meaning, ensuring that there were at least two or more like findings per category. The categories were then reviewed by the same authors to develop consensus. An explanatory statement was then developed by MC and NF that conveyed the inclusive meaning of a group of similar findings. The categories were then evaluated by the same authors, and those with commonality were aggregated into synthesised findings. As with categories, an explanatory statement was also developed, which represented conclusions based on the findings from the included studies that conveyed the inclusive meaning of a group of similar categories. All synthesised findings were reviewed by each author.

### Data integration

Integration of the quantitative and qualitative data were limited because the data were not complimentary. As a result, a narrative synthesis was conducted, which involved comparing the results from the quantitative synthesis with the qualitative synthesis and analysing the association between psychosocial factors with foot/ankle pain while considering the experiences of the participants.

## Results

### Study inclusion

**Fig. 1:**
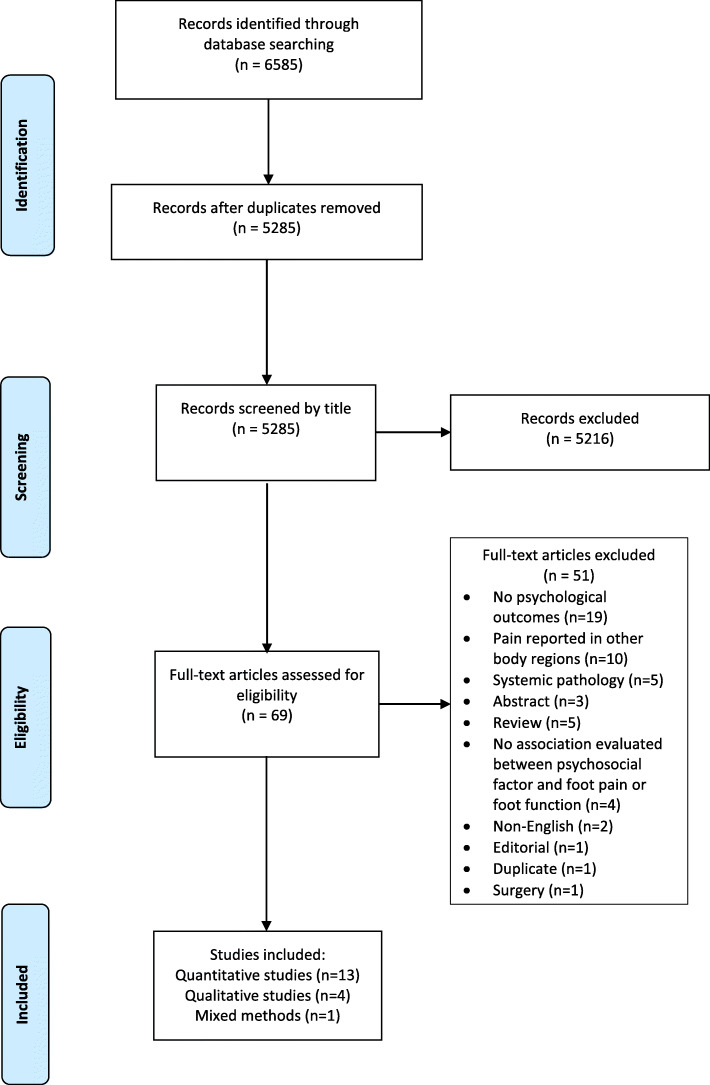
PRISMA flow diagram of included studies

A total of 6585 studies were identified through electronic databases (Fig. 1). After removal of duplicates, 5285 titles were screened. 5216 studies were subsequently excluded leaving 69 studies to be assessed for full-text retrieval and MMAT analysis. Following the quality analysis, 18 studies met the eligibility criteria and were available for analysis. Reasons for excluding 51 studies can be found in Supplementary File 3.

In total, the review included 6906 participants. Of the 18 studies included, seven were cross-sectional studies with comparative data from an asymptomatic control group [21–27]; five were cross-sectional studies without comparative data [28–32]; one was a cohort study [33]; four were qualitative studies [34–37]; and one was a mixed methods study [38]. Of the qualitative and mixed method studies, three related to Achilles tendinopathy [35, 36, 38], one to plantar heel pain [34] and one to osteoarthritis of the ankle [37].

### Methodological quality

The specific details of the MMAT quality ratings for each of the 18 studies are provided in Table 1. Of the 14 studies that included quantitative data, a ‘yes’ was awarded for the majority of the five methodological quality criteria for this category. However, 13 of the 14 quantitative studies were allocated a ‘no’ for the criterion *Are the confounders accounted for in the design and analysis?* (criterion 3.4), as they did not report the presence or absence of pain elsewhere in the body, which could have confounded the study’s reported outcome measures (i.e., psychological variables).

**Table 1:** Quality appraisal of included studies using the Mixed Methods Appraisal Tool (MMAT)

Author, Year	Screening		Qualitative studies					Quantitative non-randomised studies					Mixed methods studies					Score
	S1	S2	1.1	1.2	1.3	1.4	1.5	3.1	3.2	3.3	3.4	3.5	5.1	5.2	5.3	5.4	5.5	
**Awale 2015**	Yes	Yes						Yes	Yes	Yes	Yes	Yes						100%
**Briet 2016**	Yes	Yes						Yes	Yes	Yes	No	Yes						80%
**Butterworth 2015**	Yes	Yes						Yes	No	Yes	No	Yes						60%
**Ceravolo 2018**	Yes	Yes	Yes	Yes	Yes	Yes	Yes	Yes	Yes	Yes	No	Yes	Yes	Yes	No	No	Yes	80%
**Chimenti 2020**	Yes	Yes						Yes	Yes	Yes	No	Yes						80%
**Cotchett 2015**	Yes	Yes						Yes	Yes	Yes	No	Yes						80%
**Cotchett 2016**	Yes	Yes						Yes	Yes	Yes	No	Yes						80%
**Cotchett 2017**	Yes	Yes						Yes	Yes	Yes	No	Yes						80%
**Cotchett 2020**	Yes	Yes	Yes	Yes	Yes	Yes	Yes											100%
**Harutaichun 2020**	Yes	Yes						Yes	Yes	Yes	No	Yes						80%
**Lentz 2010**	Yes	Yes						Yes	Yes	No	No	Yes						60%
**McAuliffe 2017**	Yes	Yes	Yes	Yes	Yes	Yes	Yes											100%
**Palomo-Lopez 2020**	Yes	Yes						Yes	Yes	Yes	No	Yes						80%
**Shivarathre 2014**	Yes	Yes						Yes	Yes	Yes	No	Yes						80%
**Silbernagel 2011**	Yes	Yes						Yes	Yes	Yes	No	Yes						80%
**Tojo 2018**	Yes	Yes						Yes	Yes	Yes	No	Yes						80%
**Turner 2020**	Yes	Yes	Yes	Yes	Yes	Yes	Yes											100%
**Yeowell 2021**	Yes	Yes	Yes	Yes	Yes	Yes	Yes											100%

**Screening questions (for all studies).**

S1: Are there clear research questions?

S2: Do the collected data allow to address the research questions?

**Qualitative.**

1.1. Is the qualitative approach appropriate to answer the research question?

1.2. Are the qualitative data collection methods adequate to address the research question?

1.3. Are the findings adequately derived from the data?

1.4. Is the interpretation of results sufficiently substantiated by data?

1.5. Is there coherence between qualitative data sources, collection, analysis and interpretation?

**Quantitative non-randomised.**

3.1. Are the participants representative of the target population?

3.2. Are measurements appropriate regarding both the outcome and intervention (or exposure)?

3.3. Are there complete outcome data?

3.4. Are the confounders accounted for in the design and analysis?

3.5. During the study period, is the intervention administered (or exposure occurred) as intended?

**Mixed methods.**

5.1. Is there an adequate rationale for using a mixed methods design to address the research question?

5.2. Are the different components of the study effectively integrated to answer the research question?

5.3. Are the outputs of the integration of qualitative and quantitative components adequately interpreted?

5.4. Are divergences and inconsistencies between quantitative and qualitative results adequately addressed?

5.5. Do the different components of the study adhere to the quality criteria of each tradition of the methods involved?

The overall methodological quality of the four qualitative studies was high, with all studies receiving a ‘yes’ for all five methodological quality criteria for this category, indicating appropriate methodology and reporting of findings.

Regarding the only mixed methods study [38], a ‘yes’ was allocated for all qualitative and quantitative items, except for criterion 3.4. For the five criteria relating specifically to mixed methods studies (criteria 5.1 to 5.5.), a ‘no’ was provided for the criteria *Are the outputs of the integration of qualitative and quantitative components adequately interpreted?* (criterion 5.3) and *Are divergences and inconsistencies between quantitative and qualitative results adequately addressed?* (criterion 5.4), while all other criteria were allocated a ‘yes’.

### Participant characteristics

The characteristics of the participants included in the quantitative studies and qualitative studies are described in Table 2 and Table 3, respectively. The quantitative studies had a mean sample size of 411 (range 27 to 3321), while the qualitative studies had a mean sample size of 12. The only mixed methods study in the review included 92 participants that completed an online survey and 11 participants that participated in a focus group. Forty nine percent of participants, across all studies, were female.

**Table 2:** Characteristics of included quantitative studies

	Group with foot/ankle pain									Group without foot/ankle pain			
Author, Year	Design	Source	N (female)	Age (mean SD)	BMI (SD)	Diagnosis	Pain duration	Pain severity	Function	Source	N female	Age (mean SD)	BMI (SD)
Awale 2016	Cross-sectional	Framingham Foot Study (2002–08)	820 (543)	NR	M: 29.0 (4.7) F: 28.0 (6.0)	Foot pain	‘On most days’	If yes, categorised as ‘no’, ‘mild’, ‘moderate’ or ‘severe’ pain.	NR	Framingham Foot Study (2002–08)	2501 (1314)	NR	NR
Butterworth 2016	Cross-sectional/comparative	Community	177 (0)	68.0	28.0 (4.3)	Foot pain	NR	NR	NR	Community	619 (0)	57.0 (NR)	27.1 (3.8)
Cotchett 2016	Cross-sectional/comparative	Community	45 (22)	56.1 (12.5)	29.3 (5.5)	Plantar heel pain	10.9 (9.5)	NR	NR	Community	45 (22)	52.4 (13)	25.7 (4.4)
Chimenti 2020	Cross-sectional/comparative	Community/university sports medicine clinic	23 (15)	49.5 (10.3)	33.7 (7.8)	Achilles tendinopathy	> 3 months	BPI (median/IQR): 2.5 (1.7–3.6)	VISA-A (median/ IQR): 39 (33–59)	Community and research match.org	23 (15)	49.2 (10.6)	31.3 (5.7)
Palamo Lopez 2020	Cross-sectional/comparative	Outpatient clinic	75 (51)	Mild HV: 37.2 (19.5) Moderate HV: 59.2 (18.4) Severe HV: 68.4 (14.6)	Mild HV: 24.3 (3.3); Moderate HV: 26.9 (3.5) Severe HV: 27.9 (3.7)	Hallux valgus	Mild HV: 15.4 (SD 37.1); Moderate HV: 88.3 (55.6); Severe HV: 190.1 (160.2)	NPRS Mild HV 1.1 (1.3); Moderate HV: 6.4, (1.9); Severe HV: 7.4 (1.6)	NR	Community outpatient clinic	25 (13)	37.5 (15.2)	26.0 (4.5)
Shivarathre 2014	Cross-sectional/comparative	Specialist foot and ankle clinic	45 (28)	51.4 (range 20–70)	NR	Foot/ankle pain	> 6 months	NR	NR	Community	45 (28)	51.1 (Range: 21–67)	NR
Silbernagle 2011	Cross-sectional/comparative	Community	5 (3)	56.0 (8.0)	NR	Achilles tendinopathy	> 5 years	VISA-A: 80 (14)	VISA-A: 80 (14)	Community	22 (11)	49 (7)	NR
Briet 2016	Cross-sectional	Community (Outpatients)	88 (41)	26 (18) (Median, IQR)	24.0 (5.1) (Median, IQR)	Lateral ankle sprain	5–7 months	Ordinal scale of pain: 2.0 (3.0) (Median, IQR)	OMAS: 59 (24) (Median, IQR)	No control			
Harutaichun 2020	Cohort/descriptive	Military	270 (0)	21.5 (1.1)	23.4 (3.81)	Plantar heel pain	NR	VAS: 4.8 (2.5)	NR	No control			
Cotchett 2017	Cross-sectional	Community	36 (20)	47.3 (range, 24.0–72.0)	24.6 (12.0)	Plantar heel pain	10.3 (12.8)	FHSQ: 45.1 (20.6) VAS 61.8 (22.3)	FHSQ: 57.1 (26.4)	No control			
Cotchett 2015	Cross-sectional	Community	84 (41)	56.1 (12.2)	29.2, range 20.39–44.30	Plantar heel pain	13.6 (12.2)	FHSQ: 40.8 (21.9)	FHSQ: 49.8 (23.9)	No control			
Lentz 2010	Cross-sectional	Outpatients	85 (45)	33.1	26.6	Foot pain	*N* = 54 less than 3 months duration	VAS: 4.5 (2.0)	LEFS: 47.5 (19.5)	No control			
Tojo 2018	Survey (cross-sectional)	Nurses working in hospital	636 (569)	20–29 years: 369 (58%); 30–39 years: 133 (21%); 40–49 years: 83 (13%); 50–56 years: 48 (8%)	Underweight: 89 (14%); normal *n* = 468 (74%); overweight: 55 (9%).	Foot and ankle pain: Great toe: 88 (14%) Lesser toe: 87 (14%) Plantar forefoot: 58 (9%) Medial arch: 56 (9%) Midfoot: 99 (16%)Ankle: 62 (10%)Plantar heel: 41 (6%) Posterior heel: 47 (7%) Overall 144 (23%)	Prevalence of pain and/or discomfort of the foot in the previous month: Using SNQ:22% (*n* = 144) Using MFPDI 51% (*n* = 323)	NR	NR	No control			
Ceravolo 2018	Survey (cross-sectional)/ qualitative	Community	92 (43)	18–24 (*n* = 7); 25–34 (17); 35–44 (34); 45–54 (22); 55–64 (9); 65–74 (3)	NR	Achilles tendinopathy	13.88 (24.52)	VISA-A: 72.3 (17.7)	VISA-A: 72.3 (17.7)	No control			

Key: BPI, Brief Pain Inventory; FHSQ, Foot Health Status Questionnaire; HV, Hallux valgus; LEFS, Lower Extremity Functional Scale; MFPDI, The Manchester Foot Pain and Disability Index; NRPS, Numerical pain rating scale; NR, not reported; OMAS, Olerud-Molander Ankle Score; VAS, visual analogue acale; VISA-A, Victorian Institute of Sports Assessment – Achilles Questionnaire.

**Table 3:** Characteristics of included qualitative studies

Author, Year	Methodology	Phenomena of interest	Setting	Participants	Data collection methods
McAuliffe 2017	A qualitative, interpretive description design	To explore the lived experience of people with AT	Community dwelling, Ireland	*N* = 8 (6 male and 2 female) *N* = 7 (Midportion AT) *N* = 1 (Insertional AT) Age: Mean (SD) 40 (NS) Mean duration of symptoms = 20.5 months	Semi-structured interviews
Ceravolo 2018	Grounded theory	To explore the quality of life and lived experience of people with AT	On-line survey distributed through email and social media Focus groups: Community dwelling, Canberra, Australia	*N* = 92 (online survey) (49 male and 43 female) Mean duration of symptoms = 13.88 (SD 24.52) months Age: refer to Table 2 *N* = 11 (Focus group) Number of males and females = NS Duration of symptoms = NS	On-line survey & focus groups
Turner 2020	A qualitative, interpretive description design	To explore the lived experience of people with AT	Community dwelling, Melbourne, Australia	*N* = 15 (8 male and 7 female) *N* = 4 (Midportion AT) *N* = 11 (Insertional AT) Age: Mean (SD) 45.2 (NS) Mean duration of symptoms 8 months	Semi-structured interviews
Cotchett 2020	A qualitative, interpretive description design	To explore the lived experience of people with PHP	Community dwelling, Melbourne, Australia	*N* = 18 (12 females, 6 males) Age: Mean (SD) 58.2 (6.6) Mean duration of heel pain = 15.9 (SD 16.3) months	Semi-structured interviews
Yeowell 2021	Gadamerian hermeneutic phenomenology	To explore the experiences of people living with painful OA ankle and their views about the non-surgical management of this condition.	National Health Service orthopaedic clinic, North West England	*N* = 9 (8 males, 1 female) 54 years (range 30–70) Median duration of symptoms = 2 years (range 1–20 years	Semi-structured interviews

Key: AT: Achilles tendinopathy; PHP: Plantar heel pain; NS: not stated; SD: standard deviation

### Quantitative evidence

Below is a narrative synthesis of studies that evaluated the psychological characteristics of participants with foot/ankle pain compared to participants who were an asymptomatic control (*n* = 7), and studies that investigated the association between psychological factors and foot/ankle pain and/or foot function (*n* = 5) (Table 4).

**Table 4:** The association between psychological factors and foot/ankle pain and function

					Psychological factor	
Author, Year	Design	Diagnosis	Pain	Function	Outcome measure	Results
Awale 2016	Observational/cross sectional - comparative	Pain, aching or stiffness in the feet	If yes, categorised as no, mild, moderate or severe pain.	NR	The Center for Epidemiologic Studies Depression Scale (CES-D)	Symptoms of depression After adjusting for age and BMI, foot pain was significantly associated with self-reported symptoms of depression (men: OR 1.84 [1.36 to 2.48]; women: OR 1.93 [1.55 to 2.45])
Butterworth 2016	Observational/cross sectional - comparative	Disabling foot pain	NR*	NR	Have you ever been diagnosed as suffering depression?	Symptoms of depression After adjusting for age, education, mobility and BMI, foot pain was significantly associated with self-reported depression (OR 2.05 [95% CI 1.30 to 3.20]).
Chimenti 2020	Observational/cross sectional - comparative	Achilles tendinopathy	BPI: median/IQR: 2.5 (1.7–3.6)	VISA-A (median/ IQR): 39 (33–59	PROMIS short from 8b – depression Tampa Scale of Kinesiophobia (TSK-17) Pain Catastrophising Scale (PCS)	Symptoms of depression There was no significant difference in symptoms of depression in people with and without Achilles tendinopathy. Kinesiophobia The Achilles tendinopathy group had significantly higher TSK scores: • Group with Achilles tendinopathy: 37.2 (95% CI 34.7 to 39.7) • Group without Achilles tendinopathy: 29.6 (27.1 to 32.1) Pain catastrophising The Achilles tendinopathy group had significantly higher PCS scores: • Group with Achilles tendinopathy: 12.6 (95% CI 9.2 to 15.9) • Group without Achilles tendinopathy: 1.9 (95%CI −1.5 to 5.2)
Cotchett 2016	Observational/cross sectional - comparative	Plantar heel pain	FHSQ: 40.8 (21.9)	NR	Depression, Anxiety and Stress Scale - 21	Symptoms of depression Participants with plantar heel pain had greater levels of depression, compared to controls: • depression (mean difference = 4.4, 95% CI 2.3 to 6.5) Symptoms of anxiety Participants with plantar heel pain had greater levels of anxiety, compared to controls: • anxiety (mean difference = 2.6, 95% CI 0.9 to 4.3) Symptoms of stress Participants with plantar heel pain had greater levels of stress, compared to controls: • stress (mean difference = 4.8, 95% CI 1.9 to 7.8).
Palamo Lopez 2020	Observational/cross sectional - comparative	Hallux valgus	NPRS Mild HV 1.1 (1.3); Moderate HV: 6.4, (1.9); Severe HV: 7.4 (1.6)	NR	Tampa Scale of Kinesiophobia (TSK-11)	Kinesiophobia Participants with painful hallux valgus (Stage III and Stage IV) had significantly higher levels of kinesiophobia compared to participants without hallux valgus: Overall kinesiophobia scores: • No hallux valgus: TSK-11 = 19.6 (SD 4.8) • Stage III hallux valgus: TSK-11 = 23.6 (SD 4.6) • Stage IV hallux valgus: TSK-11 = 26.4 (SD 4.5)
Shivarathre 2014	Observational/cross sectional - comparative	Foot and ankle pain	NR	NR	Hospital Anxiety Depression Scale (HADS)	Symptoms of depression Depression was significantly higher in people with foot and ankle pain: Patient group: HADS (depression) median IQR = 3 (2,8) Control group: HADS (depression) median IQR = 2 (1,4) Symptoms of anxiety Anxiety was significantly higher in people with foot and ankle pain: Patient Group: HADS (anxiety) median IQR = 6 (3,11) Control Group: HADS (anxiety) median IQR = 4 (3,7)
Silbernagle 2011	Observational/cross sectional - comparative	Achilles tendinopathy	VISA-A: 80 (14)	VISA-A: 80 (14)	Tampa Scale of Kinesiophobia	Kinesiophobia Overall kinesiophobia scores: • Group with Achilles tendon symptoms: TSK = 26.0 (SD 6.9) • Group without Achilles tendon symptoms TSK = 28.0 (SD 5.0)
Tojo 2018	Survey/cross sectional	Foot and ankle pain: Great toe: 88 (14%); Lesser toe: 87 (14%); Plantar forefoot: 58 (9%); Medial arch: 56 (9%); Midfoot: 99 (16%); Ankle: 62 (10%); Plantar heel: 41 (6%); Posterior heel: 47 (7%); Overall 144 (23%)	NR	NR	Japanese version of the Job Content Questionnaire	Association between job strain* and foot and ankle pain Psychosocial factors, including high job strain OR 1.6 (95% CI 1.1 to 2.3) and low job control OR 1.4 (1.0 to 2.0), were associated with foot and ankle pain (pain measured using the Standardized Nordic Questionnaire. *Job strain = psychological demands score divided by job control score
Briet 2016	Observational/cross sectional	Lateral ankle sprain	Ordinal scale of pain: 2.0 (3.0) (Median, IQR)	OMAS: 59 (24) (Median, IQR)	Pain Self-Efficacy Questionnaire (PSEQ)	Association between self-efficacy and function Greater self-efficacy at baseline correlated with better ankle specific symptoms and limitations (high OMAS) at three weeks after injury (*p* = 0.017) Greater self-efficacy at baseline was significantly associated with better ankle specific symptoms and limitations (high OMAS) and explained 24% of the variability in OMAS (*R*-squared: 0.24, *p* < 0.001). Association between self-efficacy and pain intensity Greater self-efficacy at baseline correlated with lower pain intensity at follow up (*p* < 0.01).
Harutaichun 2020	Observational/Cohort	Plantar heel pain	VAS: 4.8 (2.5)	NR	Depression, Anxiety and Stress Scale - 21	Association between anxiety and pain In multiple linear regression, anxiety was associated with pain intensity (*β* = 0.41, *p* = 0.01).
Cotchett 2017	Observational/cross sectional	Plantar heel pain	FHSQ: 45.1 (20.6) VAS 61.8 (22.3)	FHSQ: 57.1 (26.4)	Tampa Scale of Kinesiophobia −17 Pain Catastrophising Scale	Association between kinesiophobia and foot function After accounting for age, sex and BMI kinesiophobia was associated with reduced self-reported foot function (*β* = − 1.60; *p* = 0.006). Association between pain catastrophising and foot function After accounting for age, sex and BMI, pain catastrophising was associated with reduced self-reported foot function (*β* = − 1.61; *p* < 0.001). Association between pain catastrophising and first step pain After accounting for age, sex and BMI, pain catastrophising was associated with first step pain (*β* = −0.93; *p* = 0.008).
Cotchett 2015	Observational/cross sectional	Plantar heel pain	FHSQ: 40.83 (21.08)	FHSQ: 49.82 (23.98)	Depression, Anxiety and Stress Scale - 21	Association between depression and heel pain and foot function After accounting for age, sex and BMI, symptoms of depression were associated with reduced self-reported foot function (*β* = − 0.53; *p* < 0.001) and foot pain (*β* = − 0.41; *p* = 0.013) In females but not males. Association between stress and heel pain and foot function After accounting for age, sex and BMI, symptoms of stress were associated with reduced self-reported foot function (β = − 0.50; *p* = 0.001) and foot pain in females but not males stress (*β* = − 0.36; *p* = 0.024).
Lentz 2010	Observational/cross sectional	Foot pain	VAS: 4.5 (2.0)	Lower Extremity Functional Scale (LEFS): 47.5 (19.5)	Tampa Scale of Kinesiophobia (TSK-11)	Association between kinesiophobia and lower extremity function In a model including age, chronicity, age, pain, ROM index, TSK-11 scores were significantly associated with lower extremity function (*β* = 0.40, *p* = 0.001).

Key: BPI, Brief Pain Inventory; FHSQ, Foot Health Status Questionnaire; HV, Hallux valgus; LEFS, Lower Extremity Functional Scale; MFPDI, The Manchester Foot Pain and Disability Index; NRPS, Numerical pain rating scale; NR, not reported; OMAS, Olerud-Molander Ankle Score; TSK: Tampa Scale of Kinesiophobia; VAS, Visual analogue scale; VISA-A, Victorian Institute of Sports Assessment – Achilles Questionnaire; HADS, Hospital Anxiety and Depression Scale

### Psychological characteristics of people with foot/ankle pain compared to participants without foot/ankle pain

#### Emotional factors

Symptoms of depression were compared with asymptomatic controls in five studies [21–23, 25, 27]. In a sample of 3321 participants, after adjusting for age and BMI, men had a 1.84 increased odds (95% CI 1.4 to 2.5) and women a 1.93 increased odds (95% CI 1.5 to 2.4) of reporting symptoms of depression compared to those without foot pain [21]. Results from a cohort of 796 community dwelling males found that foot pain was significantly associated with self-reported depression (OR 2.16 [95% CI 1.4 to 3.3]) [22]. Shivarathre et al. [25] also found that symptoms of depression were significantly higher in participants with foot and/or ankle pain. In studies that provided a specific diagnosis to explain the origin of symptoms, Cotchett et al. [23], found that participants with a diagnosis of plantar heel pain (PHP) had greater levels of depression, compared to controls (mean difference = 4.4, 95% CI 2.3 to 6.5), while Chimenti et al. [27] found no difference in self-reported symptoms of depression in a group with and without Achilles tendinopathy.

Two studies evaluated symptoms of anxiety in participants with and without pain. Shivararthre et al. [25] found that anxiety was significantly higher in a group of participants with foot/ankle pain, while Cotchett et al. [23] found that symptoms of anxiety were significantly higher in participants with PHP compared to a group without PHP (mean difference = 2.6, 95% CI 0.9 to 4.3). In the same study, analysis of a stress subscale of the DASS-21 found that symptoms of stress were significantly higher in the group with PHP compared to those without PHP (mean difference = 4.8, 95% CI 1.9 to 7.8).

#### Cognitive factors

Three studies evaluated kinesiophobia. Palomo-Lopez et al. [24] found that kinesiophobia was higher in participants with Stage II, Stage III and Stage IV hallux valgus compared to an asymptomatic group without hallux valgus, although the difference was only significant for the comparison between Stage IV hallux valgus and the asymptomatic comparator. In people with Achilles tendinopathy, there were contrasting findings from two studies. One study found no differences in kinesiophobia scores between participants with and without symptoms associated with Achilles tendinopathy [26], while another found significantly higher levels of kinesiophobia in a group with Achilles tendinopathy compared to a group without symptoms [27].

One study evaluated catastrophising in participants with and without pain. Chimenti et al. [27] found significantly higher pain catastrophising scores in a group with Achilles tendinopathy (mean 12.6, 95% CI 9.2 to 15.9) compared to a control group (mean 1.9, 95% CI −1.5 to 5.2).

#### Other psychological factors

In a survey of 636 nurses working in a teaching hospital in Japan, there was an increased odds of high job strain (OR 1.57, 95% CI 1.05 to 2.30) and low job control (OR 1.42, 95% CI 1.02 to 2.00) compared to those without foot/ankle pain.

### The association between psychological factors with foot pain and foot function

#### Emotional factors

Two studies found a significant association between depression and stress with pain in people with PHP, although Cotchett et al. [28] found that the association between depression and pain (β = − 0.41; *p* = 0.013) and stress and pain (β = − 0.36; *p* = 0.024) were significant in females and not males. In contrast, Harutaichun et al. [33] found anxiety was strongly associated with pain in Thai male conscripts with PHP (β = 0.41, *P* = 0.01), while stress (β = − 0.50; *p* = 0.001) and depression (β = − 0.53; *p* < 0.001) were also associated with foot function in females with PHP, but not males [28].

#### Cognitive factors

Cotchett et al. [29] found that kinesiophobia (β = − 1.60; *p* = 0.006) and pain catastrophising (β = − 1.61; *p* < 0.001) were significantly associated with foot function, while pain catastrophising (β = − 0.93; *p* = 0.008) was significantly associated with first step pain in people with PHP. Similarly, kinesiophobia was significantly associated with lower extremity function in participants with general foot/ankle pain (β = 0.40, *p* = 0.001) [30].

### Qualitative evidence

From one mixed methods and four qualitative studies, three synthesised findings were identified from eight categories and 52 findings. Of the five studies, three evaluated the lived experience of participants with Achilles tendinopathy [36], one focused on PHP [34] while another investigated participants with osteoarthritis of the ankle [37]. Of the three synthesised findings, two related to psychological factors associated with the participant’s lived experience (synthesised finding 1.0 and 3.0). Synthesised finding 4.0 was unrelated to psychological factors but rather focused on living with foot/ankle pain and the perceptions of the management process by health professionals. Table 5 provides a summary of the findings, illustrations, categories and synthesised findings from one mixed methods and four qualitative studies.

**Table 5:** Findings, illustrations, categories and synthesised findings of the qualitative data

Author, Year	Findings	Illustration	Categories	Synthesised findings
McAuliffe 2017	Participants described uncertainty in the causal factors associated with the condition.	“I don’t know. It’s a question people often ask. Definitely people would say to you it’s an overuse. And well I’m not doing enough for it to be overuse. That’s what wrecks me.”	1.1 Perceptions of the cause	1.0 Participants report variability but also uncertainty about factors associated with the development of Achilles tendinopathy, plantar heel pain and ankle osteoarthritis. Fear avoidance behaviours appear common and might be associated with the belief that exercise may worsen pain and cause further injury or tendon rupture.
McAuliffe 2017	Participants described mechanical factors that might associated with the condition.	“Obviously there’s some weakness there I suppose. Or some, or is it down to running style or something. Footwear yeah.”		
Turner 2020	Nearly all participants noted that over training was probably the cause of their Achilles tendinopathy.	*“*So I feel that I probably over trained. Not so much leading up to the run, it was more I didn’t recover and allow myself time to recover afterwards and I just pushed it a little bit too far.”		
Turner 2020	Some participants believed that the lack of overall fitness was the key cause for the development of Achilles tendinopathy.	“I just assume that I’ve become slightly unfit and that I always had tight muscles in my legs and it’s kind of a consequence always had tight muscles in my legs and it’s kind of a consequence of decades of not really exercising.”		
Turner 2020	There was confusion or lack of understanding as to why their condition had not resolved.	“I don’t actually know what’s going on. When I feel the pain, I mean I feel it in the base of my Achilles, but I don’t know what’s going on.”		
Cotchett 2020	Numerous causes were proposed by participants including being overweight, a change in the level of activity, standing for long periods, walking on hard, soft and uneven surfaces, and walking barefoot.	“Probably I was overdoing it. I increased my walk because I used to do around the three to four kilometres, pushing every now and then. But since I retired, I’ve been going five and at times eight (kilometres), and then walking on both hard surface and the sand.” “I just thought it was because I put on a lot of weight during that time.” “Well I really don’t know. I didn’t understand what it was. I knew it had something to do with the plantar fascia.”		
Ceravolo 2018	Some participants were fearful of re-injury. Which prevented wanting to exercise.	“There is the fear of it reoccurring ... the fear of triggering an attack prevents you from doing stuff.”	1.2 Activity beliefs	
McAuliffe 2017	Participants noted that they were fearful of doing further damage (e.g., tear) if they continued to exercise and or failed to complete their exercise program.	“Yeah it could like. It probably ... I’d be thinking that like. By running on it as it is, I think it would. It might get to the stage where it will rupture ... If I don’t get it fixed like.”		
Turner 2020	Nearly all participants reported having significantly reduced or completely ceased certain physical activities due to fear of further injury, damage, and/or pain.	“I often just pull out earlier then. I never let it get that bad, if you know what I mean? I don’t really go in as hard. I’ve got that kind of doubt niggling in the back of my mind about it. That I need to protect it, rather than let it get too bad. So I’m not someone who would take it that far to the edge. I think that’s probably more of it, it just hinders me from going further or harder, or any of those things really.”		
Yeowell 2021	Participants described fearing the experience of pain if they undertook physical activity.	“I can get away with doing it, it’s afterwards, when I stop, it doesn’t, it’s endless... but wow the pain I felt over the following days. It’s like we’d go to the park and I don’t want to risk it. I don’t want to risk it and then it affect me; not moving tomorrow.” “If I want to play golf I just go out in a buggy [golf buggy to avoid walking], but you do a lot of twisting … … …”		
Ceravolo 2018	Participants described variable experiences related to living with their Achilles tendinopathy. Some were resigned to living with it.	“I expect it’s something I’ll be managing forever, by the sounds of it, from those people who seem to have it.” “So I just got back to that stage where I’m like—yeah, it’s almost like accepting you’ve got cancer.”	2.0 Living with the condition	
Ceravolo 2018	Some participants reported minimal impact on quality of life.	“An Achilles is not an epic thing where you can’t live a normal life or anything like that; I can’t say that it overly affected my quality-of-life.”		
Ceravolo 2018	Participants previous experiences and beliefs about their condition influenced their coping mechanism and approach.	“The attitude in my case, because having 40 years of chronic back pain off and on, you tend to say absorb it into the normality of things … Adopt a more practical and stoic approach to it I think.”		
Turner 2020	Nearly all participants reported that the Achilles tendinopathy impacted on their running, either having reduced or completely stopped running.	“It just means altering, I guess, my training regime that I’m used to, to kind of fit in with the injury. So, when there’s the running aspect in that, I just don’t even bother trying anymore.”		
Turner 2020	Those who expressed poor prognosis stated chronicity, pathology, and genetic disposition as reasons.	“It can be relieved, but there’s nothing you can do about flat feet. I’m born like that. That’s how life is. I’m not gonna [sic] play tennis again. I’m not sure that I’ll be able to play table tennis.”		
Turner 2020	Participants stated they were motivated to seek treatment for their pain and fear of disability and further deterioration of both the condition and physical activity.	“And there are still things I want to do in the future, like with running and more marathons, … ... So, I don’t have a choice but to keep it strong and keep doing those exercises.”		
Ceravolo 2018	Participants noted having to adapt their lifestyle due to their Achilles tendinopathy which impacted on social connections.	“There are things that you can do, whereas I don’t know about you guys, but when I had the Achilles, it was like being in jail, that real restriction of your freedom for things that you enjoy doing.”	3.1 Impact on activity and participation	3.0 The impact of Achilles tendinopathy, plantar heel pain and osteoarthritis of the ankle is individual presenting with emotional, physical challenges and a loss of self.
Turner 2020	All participants reported that their daily routines and activities were affected by their Achilles tendinopathy.	“I think it restricts me in a lot of things that I would be able to do. I don’t think I can go out and kick the footy with my son, or... You know, I manage to... in pain, to go for a walk with the dog in the evening, if you know what I mean?”		
Turner 2020	Nearly all the participants reported having reduced or completely stopped running due to their Achilles tendinopathy.	“It just means altering, I guess, my training regime that I’m used to, to kind of fit in with the injury. So, when there’s the running aspect in that, I just don’t even bother trying anymore.”		
McAuliffe 2017	Some participants reported disruption at work, depending on the demands of work.	“Aww, it’s just when you have to go anywhere, walk around the office, walking to go get a coffee. You get up; you’ve forgotten about it sitting down … once you get up it’s like ouch. Then you’re almost limping everywhere. It’s noticeable enough in that people would say are you all right, what are you limping for.”		
McAuliffe 2017	The physical disruption varied based on participants symptoms, however morning pain was more common.	“But in the morning times in particular, very sore in the mornings. You’re literally hobbling around the place until you get moving as such.”		
Cotchett 2020	Most participants described a negative impact on physical function.	“I don’t feel as strong in my whole body. I have a bit more trouble lifting, trouble walking, especially down slopes or downstairs, not so bad going up stairs or up slopes but activity has certainly been slowed right down.”		
Yeowell 2021	The symptoms experienced impacted on participants function and social life.	“I get no enjoyment out of it [dancing] like I used to. and that means I’ve stopped doing that, because I’m not sufficiently ‘macho’ that I will force myself to do it if it hurts.” “We tend to go the climbing centre and I’m very limited to where I can go. My friend was into his hiking and we went on loads of walks and I just can’t go.” “I’m sat in the house, I can’t do nothing. I have no social life. My friends, they all say come and have a pint, but what’s the good in going for a pint when I’m sat there, I can’t move, I can’t go to the bar, I can’t get to the toilet.” “… I’m not living well with this right ankle. It’s just stopping the living of life, I can’t live my life with it, it’s crazy.”		
Yeowell 2021	Negative impact on strength and stability.	“My ankle just doesn’t feel strong. I don’t like walking on cobbles or uneven surfaces as it feels I will go over on my ankle.”		
Ceravolo 2018	Some participants noted the negative impact on their mental health including feeling depressed, stressed and reduced self-esteem.	“I don’t want to overstate the cranky and anger stuff, but there’s definitely a general feeling of—it’s almost depression, but not clinical depression, but you just don’t feel good about yourself or the world.”	3.2 Pain impacts emotions	
Ceravolo 2018	Achilles tendinopathy limited exercise which negatively impacted on mental health. Exercise relieves stress and increases energy.	“So I rely on exercise heavily to maintain my mental health. The endorphins that I get from training are vital to my mental well- being. To not be able to train would be a devastating impact I think on my mental health.”		
Turner 2020	A majority of participants described frustration and/or annoyance with their condition and its limitations on activity.	“Well, I think it’s just like there’s things that I enjoy doing and if I can’t do them, now I get a bit frustrated and it’s part of what makes me happy and makes me satisfied with things. Yeah, I think it’s part of those basic sort of … You know, you do a nice, long run and you feel quite good after it. I’m not having that experience. I think that satisfaction, the challenge, and all that sort of stuff, I’m just not being able to do and expose myself to and I kind of struggle to find that in other modes when I’m not running.” “It’s disappointing and it’s pretty frustrating, really, because it feels like it’s something that will never go away, but yeah, it’s just very frustrating is probably the biggest thing, really.”		
Turner 2020	There was a feeling of a lack of control over their condition.	“I no longer feel that I am in control now.” “I failed to go to the gym this morning, because I was feeling fed up with myself and so I’m not really in control of any of these things.”		
Cotchett 2020	A reduction in physical activity was associated with negative emotions and feelings including hopelessness and frustration.	“I’m a mouse on a wheel. I can’t seem to get off. I don’t know what to do. I don’t know how to lose weight without moving, and how do you move without the pain? So yeah, sometimes I’m a bit exasperated by it”. “But honestly, if someone told me to mix up a special drink cause that’s what was gonna fix it that’s probably what I’d do as well. So, I probably am trying anything. It’s a bit like spin the wheel and try your luck. I’ll try it all.”		
Yeowell 2021	Participants described a negative impact on mood due to their experience of ankle osteoarthritis.	“I got quite depressed with it all. I didn’t realise that there was such an adverse effect that the pain grinds you down and gives you that low self-esteem and no self-confidence. You can see other people your age doing things but you’re not able to. It wears you down mentally and makes you very depressed at times. What you don’t realise is it’s not just physical, it very much affects you mentally.” “It’s just always there and it just hurts. If it was just one or two blasts of tooth ache then you’d be ok; “ooh that’s not nice”, but when it’s there every time you walk, it just gets you down.”		
Yeowell 2021	Pain is the central issue.	“The pain is horrific. It’s just terrible, I wish someone could. .. you know what, I would have it cut off and a false one there if they could. It’s horrendous pain, it’s terrible. It’s driving me round the bend. I’d go for anything to get rid of this pain, I’d try anything now.”		
Ceravolo 2021	Participants who identified as physically fit described a negative impact on self-image and self-esteem. Quality of life appeared to be more negatively impacted for those whose self-image was affected.	“I’d also say that, in some respects, it (running) defines who I am, and so, if I can’t do that, it’s taking who I am away from me. That’s tough... Well the self-esteem certainly goes down, because you’re not who you were.”	3.3 Loss of self	
McAuliffe 2017	Participants noted a negative effect on the ability to undertake activities that are enjoyable and contribute to their social life and sense of self.	“Seeing my peers going to races, winning races or getting PBs. Progressing ... and I’m stuck here. That has been horrendous I have to say. Now, I know there are worse things in life that can happen. But it’s been horrible.”		
Yeowell 2021	Participants felt that they had experienced a change in their self-identity and perceived this as a loss of self-worth.	“I was like the leader, and them the handbrakes because they were slower than me; now I’m the handbrake.” “I’m just useless, just because of a daft ankle. It’s unbelievable that isn’t it. It makes me feel as if I’m good for nothing, I might as well just turn it in, you know, just go for a couple of tablets and I’ll call it a day. Just a waste of time. I’m good for nothing at the minute. I feel like crying. It’s horrible. Every day of my life; it gets a bit upsetting. You just wanna give in, in the end, you get sick of it.” “…. I didn’t realise that there was such an adverse effect that the pain grinds you down and gives you that low self-esteem and no self-confidence … ..”		
McAuliffe 2017	Participants noted frustration with the treatment process and the uncertainty of professional opinion.	“For me there were a lot of grey areas. Everybody was giving me, like telling me different things. So like nobody knew 100% everything about Achilles and it was varying, you know people opinions were varying.”	4.1 Perceptions of the management process by health professionals	4.0 External factors from health professionals can affect participants’ experience of Achilles tendinopathy, plantar heel pain and ankle arthritis. Participants described unmet needs regarding a clear diagnosis and management plan. Opinions from health professionals were often conflicting or lacking in detail to obtain an understanding of the condition and the treatment pathway or options. Exercise (strengthening) was considered important although adherence was problematic for people with Achilles tendinopathy. Passive treatments were considered important for positive treatment outcomes in people with plantar heel pain.
Ceravolo 2018	Participants previous experiences and beliefs about their condition influenced their decision on treatment options.	“So they tell you never to stop activity, but I think you really need to do that with Achilles.”		
Turner 2020	Frustrations and/or dissatisfaction with healthcare providers and the education they received was a common theme and was reported by several participants.	“Sometimes when you talk to your doctor, or the specialist, it’s very limited time, and they don’t have the time to explain it properly, and they speak in technical terms. I thought the physio spent a bit more time with you to talk to you about it.”		
Cotchett 2020	Gathering information from various sources to understand and improve PHP was variable.	“I did actually go to the doctor once and described it to him, but – yeah, he just said massage the foot. The GP didn’t look in the slightest bit interested, really.”		
Cotchett 2020	Needing better understanding of PHP, and seeking better management.	“If I had a better idea, better understanding of what was actually happening with it. I think that would have guided me a lot better in what I was doing about it.”		
Cotchett 2020	Need for a better explanation and justification of the treatment plan.	“A lot of explanation is being well “Do this or do that” but without really explaining what it is that you’re doing and why you’re doing it and what you’re supposed to observe.”		
Cotchett 2020	Participants expressed frustration with conflicting information regarding the best approach to manage PHP with a spectrum of messages being delivered by clinicians and resources available online.	“I mean there’s so much conflicting information on the internet, trying to put that into context with what doctors told me, what my physio friend at work told me and reading different things on the internet, trying to relate that to my condition, and work it all out” (Participant 3).		
Cotchett 2020	A strong theme emerged regarding the importance of seeking early diagnosis and advice from a health professional.	“Get a good diagnosis and someone who knows what they’re doing. I suppose looking back, getting a firmer diagnosis at the beginning maybe, trying to get some information that seemed to relate more specifically to your own condition because I found that I was never really quite clear on what advice I was getting really related to what I had because of confusion over what it was” (Participant 3).		
Yeowell 2021	Participants described not being taken seriously in the early stages of the disease.	“They x-rayed it, and they said it’s not too bad. They said you can see it, but it’s not bad. .. but I can barely walk on it [in the mornings] until it wakes itself up a bit.” “In some sense, it seems that it’s not being treated seriously, and if I physically couldn’t walk, I’d be referred to people. Actually, it matters. Long before people are physically incapacitated, because it’s affecting my mental health, it’s affecting my physical health, but it’s not extreme enough with the level of funding the NHS has at the moment for it to be treated seriously. With ankle pain, the pain doesn’t have to be that bad to have a massive impact on your quality of life.”		
Turner 2020	Nearly all of the participants were prescribed or sought passive treatment for management of their Achilles tendinopathy.	“So, acupuncture tends to... I respond really well to that and pretty quickly as well. Obviously, massage, anything to loosen up my calf, really. So, massage work or acupuncture on my calves.”	4.2 Individual experiences with treatment.	
Turner 2020	Several of the participants believed strength training was the most effective treatment in Achilles tendinopathy.	“I failed to really maintain it properly. I should have done more of those exercises prescribed.”		
McAuliffe 2017	Participants expressed the belief that forceful passive treatments were required.	“He wore a kind of a knuckle duster and really rubbed them hard. And I think that’s the only thing that got rid of the morning stiffness.”		
McAuliffe 2017	Participants noted the expectation of exercise but were either non-adherent or did not find the principle plausible.	“There was a therapy that was recommended to me maybe at the start of the year, it was called heel drops or painful heel drops? They’re on the edge of the steps and you basically flex, you basically flex down and flex up ... Was it 10 min a night every night for 12 weeks but in my head that was crazy.” “There was a therapy that was recommended to me maybe at the start of the year, it was called heel drops or painful heel drops? They’re on the edge of the steps and you basically flex, you basically flex down and flex up ... Was it 10 min a night every night for 12 weeks but in my head that was crazy.”		
Cotchett 2020	Prefabricated foot orthoses had been explored by participants but with mixed success.	“Well, talk about painful, they were dreadful and then most of my shoes, I couldn’t wear because my instep is too high, and I couldn’t get the foot in the shoe with the orthotics in it” (Participant 5). “The thing I’ve had the most success with is some orthotic inner soles that are very rigid and seem to hold my foot still. That seems to give me the most support” (Participant 18).		
Cotchett 2020	Footwear played a key role in alleviating pain with participants highlighting the importance of wearing supportive shoes with a small degree of heel elevation, and caution was expressed when walking in flat unsupportive shoes.	“Go invest in a good pair of shoes firstly. Never walk barefoot or in thongs and even if you get up in the middle of the night, make sure you put a shoe on to walk around” (Participant 7).		
Cotchett 2020	Tension existed in participants’ responses relating to the role of modifying activity and or rest. While some participants modified their behaviour by limiting or eliminating an activity, others questioned the importance of rest.	“I didn’t realise that this would help but I’ve started running and I’ve stopped eating sugar and I don’t think the sugar has a relationship but what it has done is help me lose weight and get healthy” (Participant 17). “Maybe I just didn’t give it long enough, but I did have a week of total rest and it didn’t help my foot and my brain nearly went into a massive meltdown” (Participant 18).		
McAuliffe 2017	Participants noted that rest was often a treatment decision made by the health professional or the patient themselves although there were conflicting opinions about the value of rest.	“I’ve gone to a lot of physios and they tell me it’s an overuse injury but I don’t think it is ‘cause I’ve stopped. People say stop and don’t do anything for 6 or 8 weeks but if I did stop and went back even for a jog a couple of miles after not doing anything for a period of time id still be in pain like it never stopped.”		
Yeowell 2021	Mixed experiences with non-surgical management.	“It was gentle exercises, which helped the stiffness in the joint. Doing any kind of mild exercise was unbelievable and the manipulation stuff helped because it kept the joints from freezing up. So, they definitely helped.” “I have an ankle brace, it’s really good. .. off course in the summer time they’re not great..That’s because it is hot and your feet start sweating more. You get sores on your feet with them, so I take them off.“ “The physio gave me some exercises which, quite frankly, didn’t really help. .. it was just movement exercises and strengthening – the idea being to strengthen the joint.”		

#### Synthesised finding 1.0. Participants report variability and uncertainty about factors associated with the development of Achilles tendinopathy, PHP and osteoarthritis of the ankle. Fear avoidance behaviours appear common and might be associated with the belief that exercise may worsen pain and cause further injury or tendon rupture

This synthesised finding was derived from two categories, one of which related to psychological factors associated with the participants’ experience of Achilles tendinopathy and ankle osteoarthritis.

##### Category 1.2. Activity beliefs

Participants reported having significantly modified or ceased their activity because of a fear of further injury, damage and/or an increase in pain:

*“I often just pull out earlier then. I never let it get that bad, if you know what I mean? I don’t really go in as hard. I’ve got that kind of doubt niggling in the back of my mind about it. That I need to protect it, rather than let it get too bad. So I’m not someone who would take it that far to the edge. I think that’s probably more of it, it just hinders me from going further or harder, or any of those things really” (participant with Achilles tendinopathy)* [35]*.*

*“There is the fear of it reoccurring ... the fear of triggering an attack prevents you from doing stuff” (participant with Achilles tendinopathy)* [38]*.*

“*I can get away with doing it, it’s afterwards, when I stop, it doesn’t, it’s endless... but wow the pain I felt over the following days. It’s like we’d go to the park and I don’t want to risk it. I don’t want to risk it and then it affect me; not moving tomorrow” (participant with osteoarthritis of the ankle)”* [37].

#### Synthesised finding 3.0. The impact of Achilles tendinopathy, PHP and osteoarthritis of the ankle is individual, presenting with emotional, physical challenges and a loss of self

The synthesised finding was derived from three categories, two of which related to psychological factors.

#### Category 3.2. Pain impacts on emotions

Some participants with Achilles tendinopathy noted the negative impact on their mental health including feeling depressed, stressed and a sense of hopelessness:

*“I don’t want to overstate the cranky and anger stuff, but there’s definitely a general feeling of—it’s almost depression, but not clinical depression, but you just don’t feel good about yourself or the world” (participant with Achilles tendinopathy)* [38]*.*

*“I’m a mouse on a wheel. I can’t seem to get off. I don’t know what to do. I don’t know how to lose weight without moving, and how do you move without the pain? So yeah, sometimes I’m a bit exasperated by it” (participant with PHP)* [34]*.*

*“I got quite depressed with it all. I didn’t realise that there was such an adverse effect that the pain grinds you down and gives you that low self-esteem and no self-confidence. You can see other people your age doing things but you’re not able to. It wears you down mentally and makes you very depressed at times. What you don’t realise is it’s not just physical, it very much affects you mentally” (participant with osteoarthritis of the ankle)* [37]*.*

In addition, participants described frustration and/or annoyance with their condition and management:

*“Well, I think it’s just like there’s things that I enjoy doing and if I can’t do them, now I get a bit frustrated and it’s part of what makes me happy and makes me satisfied with things” (participant with Achilles tendinopathy)* [35]*.*

*“But honestly, if someone told me to mix up a special drink cause that’s what was gonna fix it that’s probably what I’d do as well. So, I probably am trying anything. It’s a bit like spin the wheel and try your luck. I’ll try it all” (participant with plantar heel pain)* [34]*.*

#### Category 3.3. Loss of self

Participants with Achilles tendinopathy and osteoarthritis of the ankle described a negative impact on self-image and self-esteem:

*“I’d also say that, in some respects, it (running) defines who I am, and so, if I can’t do that, it’s taking who I am away from me. That’s tough... Well the self-esteem certainly goes down, because you’re not who you were” (participant with Achilles tendinopathy)* [38]*.*

*“Seeing my peers going to races, winning races or getting PBs. Progressing ... and I’m stuck here. That has been horrendous I have to say. Now, I know there are worse things in life that can happen. But it’s been horrible” (participant with Achilles tendinopathy)* [36]*.*

*“I’m just useless, just because of a daft ankle. It’s unbelievable that isn’t it. It makes me feel as if I’m good for nothing, I might as well just turn it in, you know, just go for a couple of tablets and I’ll call it a day. Just a waste of time. I’m good for nothing at the minute. I feel like crying. It’s horrible. Every day of my life; it gets a bit upsetting. You just wanna give in, in the end, you get sick of it” (participant with osteoarthritis of the ankle)* [37]*.*

*“… I didn’t realise that there was such an adverse effect that the pain grinds you down and gives you that low self-esteem and no self-confidence …”(participant with osteoarthritis of the ankle)* [37]*.*

### Integration of quantitative evidence and qualitative evidence

Integration of the quantitative and qualitative data were not possible due to the disparate nature of the evidence available. More specifically, the majority of the qualitative data related to the lived experience of participants with Achilles tendinopathy, which might not directly relate to other musculoskeletal conditions explored in the quantitative review such as PHP, hallux valgus, ankle sprains and general foot pain. As such, a narrative synthesis was undertaken.

Despite the disparate evidence, the results from individual syntheses were mostly supportive. The quantitative data, which highlighted that the emotional [21–23, 25] and cognitive [24] state of participants with foot/ankle pain was different to people without foot and/or ankle pain, might be explained by elements of the qualitative data. Most participants with Achilles tendinopathy, PHP and osteoarthritis of the ankle described frustration associated with their experience of pain and the inability to undertake activities that brought enjoyment, often leading to a loss of self, a loss of social connections and an inability to manage emotions due to pain and functional limitations. For some participants, frustration may be associated with emotional distress due to feelings of despair, helplessness, pain-related fear and unfulfilled needs, which might be due to the uncertainty of the diagnosis, management, and prognosis of the condition. Some participants were clearly frustrated and dissatisfied with the treatment process offered by health professionals, including the uncertainty of professional opinion and the type and level of education received.

There were some aspects of the quantitative evidence that were not directly explored in the qualitative studies. For example, pain catastrophising was significantly higher in people with foot/ankle pain compared to those without foot/ankle pain and was associated with increased pain severity and poor function in people with PHP, however the domains relating to magnification, rumination and helplessness were not explored in the qualitative studies. In addition, the quantitative evidence found that symptoms of depression were associated with the presence of foot/ankle pain, although the qualitative evidence did not provide a deep or rich exploration of these symptoms in people with Achilles tendinopathy or PHP. However, a few participants with osteoarthritis of the ankle described a substantial impact of their condition on their emotional state. There were also several aspects of the qualitative data that were not explored in the quantitative studies. For example, a loss of self was evident in the studies by McAuliffe et al. [36] and Turner et al. [35] although this was not a variable that was evaluated in the quantitative studies.

## Discussion

The purpose of this mixed methods systematic review was to evaluate and understand the psychological characteristics of people with foot/ankle pain. A comprehensive search of the evidence found 18 studies that met the inclusion criteria using quantitative, qualitative and mixed methods designs. A range of conditions were explored including non-specific foot pain, PHP, Achilles tendinopathy, painful hallux valgus and osteoarthritis of the ankle. The overall methodological quality of the studies was rated as high, although the quantitative findings were largely derived from observational cross-sectional studies, which reduces the certainty of the evidence. The review identified negative psychological characteristics of participants with foot/ankle pain that differed from participants without pain, and an association between negative psychological factors with foot pain and foot function for certain conditions. In addition, qualitative findings revealed that the experience of pain has a negative impact on emotions and function, is associated with pain-related fear, but also a loss of self. Due to the disparate nature of the evidence available, integration of the quantitative and qualitative data were not possible.

### Psychological factors in participants with and without foot/ankle pain

Four of seven cross-sectional studies reported increased symptoms of depression and anxiety in participants with foot/ankle pain compared to those without. This finding is consistent with other musculoskeletal conditions such as patellofemoral pain [39], knee arthroplasty [40] and back pain [41]. However, despite the perceived association between symptoms of emotional distress and foot/ankle pain, the results have largely been derived from cross sectional studies that had limited statistical control over confounding factors that might be associated with the outcome. In addition, the clinical relevance of the difference in psychological features between people with and without foot/ankle pain is uncertain due to the wide range of psychological measures that were used to evaluate emotional distress, preventing data from being pooled.

### Psychological factors associated with foot pain and foot function

Two studies found a significant association between emotional distress with foot pain and foot function in people with PHP, although regression analyses found contrasting findings. Cotchett et al. [29] found a significant association between depression and pain and stress and pain in females (but not males), while anxiety was not associated with pain in males or females with PHP. In contrast, Harutaichun et al. [33] found (using the same outcome measure) a significant association between anxiety and pain among male Thai conscripts. The contrasting findings may be explained by differences between the samples. The male Thai participants were younger and had a substantially higher anxiety level compared to participants in the Cotchett et al. [29] study, which might be related to performance demands and the continuing physical threat associated with being a Thai conscript in the military [42].

Cotchett et al. [29] also found that kinesiophobia and pain catastrophising were significantly associated with foot function, and pain catastrophising was significantly associated with first step pain in people with PHP. Similarly, kinesiophobia was significantly associated with lower extremity function in participants with general foot/ankle pain. These findings are consistent with other research that found kinesiophobia has a moderate positive relationship with disability [43], and pain catastrophising is associated with pain in other musculoskeletal conditions [44–46].

### The lived experience of people with foot/ankle pain

The four qualitative and one mixed methods study provided rich data about cognitive factors associated with foot/ankle pain but also the emotional impact and a loss of self. Descriptions of fear avoidance behaviours were common and might stem from the belief that exercise may worsen pain and cause further injury or tendon rupture. However, the impact of Achilles tendinopathy, PHP and osteoarthritis of the ankle was individual with some participants presenting with emotional distress, physical challenges, and a loss of self. These findings are consistent with other musculoskeletal conditions such as knee osteoarthritis [47], patellofemoral pain [48], low back [49], and shoulder pain [50], and highlights the importance of listening to and understanding the patient’s perspectives and context related to their experience of pain.

### Gender disparities related to psychological factors and pain

In studies that reported data from females and males separately, symptoms of depression were higher in females compared to males [21] [23], which is consistent with the prevalence of depression amongst females and males in the wider community [51].

In studies where the severity of foot pain was stratified by gender, females reported higher levels of general foot/ankle pain [21] and also PHP compared to male counterparts [28]. This disparity is also consistent with the experience of pain for females and males in the community. Research has indicated that females with osteoarthritis of the knee experience more widespread pain, increase frequency of daily pain, and increased pain related to affective symptoms compared to males [52].

### Strengths and limitations

There are several strengths of this review which include pre-registration with PROSPERO, reporting according to the PRISMA guidelines and adhering to the JBI approach to Mixed Methods Systematic Reviews. However, this review should be considered in light of some limitations. The review is limited by the small number of studies that evaluated participants over time, so it is not possible to evaluate the temporal relationships between psychological factors and foot/ankle pain. In addition, cross-sectional studies are subject to selection bias introduced by the study investigator or the participant themselves [53]. Confounding might be an issue for many of the cross-sectional studies as other factors known to be associated with pain or function were not attended to, which might reduce the validity of the true association between the psychological variable and the outcome of interest [54].

### Recommendations for practice

Due to the cross-sectional nature of most of the included studies, caution is required when making recommendations for practice. However, there is growing evidence that psychological factors are associated with the experience of pain including the development and maintenance of persistent musculoskeletal pain, and therefore should be assessed in people with foot/ankle pain [10].

The psychological factors reported in this review are not routinely assessed by many health professionals who treat musculoskeletal pain. This may be due to inadequate training and/or the inability to apply theory to practice [10]. There is clearly a need for better screening of psychological risk factors to identify patients who may be at risk of poor clinical outcomes. Screening can first occur during the patient interview to understand the patient’s context but also their perspective of their experience of pain and disability. The interview provides the framework for screening of psychological factors by using a multidimensional screening tool (e.g., Orebro Musculoskeletal Pain Screening Questionnaire) [55] to identify people at a high risk of a poor outcome due to psychological factors. If appropriate, a unidimensional measure can then be used to specifically identify psychological factors to enhance clinical reasoning and implement a psychologically informed treatment.

### Recommendations for research

There is clearly enough evidence from cross-sectional studies to support an association between psychological factors and foot/ankle pain. However, the direction of the relationship needs to be determined through longitudinal studies to explore the temporal relationship between pain and psychological factors. In addition, there is a need for more mixed methods studies so that quantitative data can be used to explain findings from qualitative data, but also the qualitative inquiry can help develop hypotheses for testing in the quantitative component. Future studies could consider exploring in more depth the source and impact of frustration in people with foot/ankle pain, which might be amenable to treatment by health professionals through acknowledgement, reassurance, and better communication. Future research should determine the predictive ability of multidimensional screening tools in identifying patients at risk of developing persistent foot/ankle pain. Finally, a large number of studies were excluded during the screening process as a specific psychological factor was not evaluated. Rather, many studies evaluated health related quality of life. While such generic measures are important to evaluate multiple components such as an individual’s physical health, mental, and social relationships future research should consider supplementing these measures with disease-specific measures to address clinically important positive and negative changes in psychological factors.

## Conclusions

This review provides evidence that negative psychological constructs are higher in participants with foot/ankle pain compared to those without foot/ankle pain, and an association between negative psychological factors with foot pain and foot function for certain conditions. In addition, the experience of pain has a negative impact on emotions and function, is associated with pain-related fear and a loss of self. However, the cross-sectional nature of the study designs included in this review reduce the certainty of the evidence. The review highlights the importance of assessing foot-related musculoskeletal pain through a biopsychosocial model lens.

## Supplementary Information

**Additional file 1:.** Supplementary file 1 – Medline database search strategy (03/03/2021).

**Additional file 2:.** JBI QARI Data Extraction Tool for Qualitative Research_Ceravolo (Data extracted by Frescos).

**Additional file 3:.** Supplementary file 2 – List of excluded studies.

## Data Availability

Not applicable.
