# Supplementary material for: Psychological factors associated with foot and ankle pain: a mixed methods systematic review
Source: J Foot Ankle Res. 2022 Feb 3;15:10. doi: 10.1186/s13047-021-00506-3 (PMC8812226; doi:10.1186/s13047-021-00506-3)
Supplement: Supplementary file 1 — Additional file 1:. Supplementary file 1 – Medline database search strategy (03/03/2021). [file 13047_2021_506_MOESM1_ESM.docx]

**Supplementary file 1 – Medline database search strategy (03/03/2021)**

1. exp Psychology/

2. psychosocial.mp. [mp=title, abstract, original title, name of substance word, subject heading word, floating sub-heading word, keyword heading word, organism supplementary concept word, protocol supplementary concept word, rare disease supplementary concept word, unique identifier, synonyms]

3. psycho-social.mp. [mp=title, abstract, original title, name of substance word, subject heading word, floating sub-heading word, keyword heading word, organism supplementary concept word, protocol supplementary concept word, rare disease supplementary concept word, unique identifier, synonyms]

4. biopsychosocial.mp. [mp=title, abstract, original title, name of substance word, subject heading word, floating sub-heading word, keyword heading word, organism supplementary concept word, protocol supplementary concept word, rare disease supplementary concept word, unique identifier, synonyms]

5. bio-psychosocial.mp. [mp=title, abstract, original title, name of substance word, subject heading word, floating sub-heading word, keyword heading word, organism supplementary concept word, protocol supplementary concept word, rare disease supplementary concept word, unique identifier, synonyms]

6. biopsycho-social.mp. [mp=title, abstract, original title, name of substance word, subject heading word, floating sub-heading word, keyword heading word, organism supplementary concept word, protocol supplementary concept word, rare disease supplementary concept word, unique identifier, synonyms]

7. bio-psycho-social.mp. [mp=title, abstract, original title, name of substance word, subject heading word, floating sub-heading word, keyword heading word, organism supplementary concept word, protocol supplementary concept word, rare disease supplementary concept word, unique identifier, synonyms]

8. psycholog*.mp.

9. emotion*.mp.

10. exp Depression/

11. depression.mp. or Depression/

12. depressive.mp.

13. exp Stress, Psychological/

14. stress.mp.

15. exp Anxiety/

16. anxiety.mp. or Anxiety/

17. exp Catastrophization/

18. catastrophi*.mp.

19. exp Cognition/

20. cognitiv*.mp.

21. exp Self Efficacy/

22. self-efficacy.mp.

23. Hypervigilan*.mp. [mp=title, abstract, original title, name of substance word, subject heading word, floating sub-heading word, keyword heading word, organism supplementary concept word, protocol supplementary concept word, rare disease supplementary concept word, unique identifier, synonyms]

24. (Fear* adj2 pain*).mp. [mp=title, abstract, original title, name of substance word, subject heading word, floating sub-heading word, keyword heading word, organism supplementary concept word, protocol supplementary concept word, rare disease supplementary concept word, unique identifier, synonyms]

25. (Fear* adj2 (activit* or movement* or avoidance)).mp. [mp=title, abstract, original title, name of substance word, subject heading word, floating sub-heading word, keyword heading word, organism supplementary concept word, protocol supplementary concept word, rare disease supplementary concept word, unique identifier, synonyms]

26. (Avoidance adj2 (activit* or movement* or pattern* or behaviour* or behavior*)).mp. [mp=title, abstract, original title, name of substance word, subject heading word, floating sub-heading word, keyword heading word, organism supplementary concept word, protocol supplementary concept word, rare disease supplementary concept word, unique identifier, synonyms]

27. kinesiophobia.mp. [mp=title, abstract, original title, name of substance word, subject heading word, floating sub-heading word, keyword heading word, organism supplementary concept word, protocol supplementary concept word, rare disease supplementary concept word, unique identifier, synonyms]

28. coping.mp. [mp=title, abstract, original title, name of substance word, subject heading word, floating sub-heading word, keyword heading word, organism supplementary concept word, protocol supplementary concept word, rare disease supplementary concept word, unique identifier, synonyms]

29. ((plantar or foot or feet or toe or hallux or arch or ankle or heel or forefoot or midfoot or rearfoot or subtalar joint or midtarsal joint or metatarsophalangeal or achilles or triceps surae) adj3 (pain* or inflam* or tendinitis or tendonitis or tendinopath* or tendonopath* or sprain*)).mp. [mp=title, abstract, original title, name of substance word, subject heading word, floating sub-heading word, keyword heading word, organism supplementary concept word, protocol supplementary concept word, rare disease supplementary concept word, unique identifier, synonyms]

30. exp Fasciitis, Plantar/

31. plantar heel pain.mp. [mp=title, abstract, original title, name of substance word, subject heading word, floating sub-heading word, keyword heading word, organism supplementary concept word, protocol supplementary concept word, rare disease supplementary concept word, unique identifier, synonyms]

32. 1 or 2 or 3 or 4 or 5 or 6 or 7 or 8 or 9 or 10 or 11 or 12 or 13 or 14 or 15 or 16 or 17 or 18 or 19 or 20 or 21 or 22 or 23 or 24 or 25 or 26 or 27 or 28

33. 29 or 30 or 31

34. 32 and 33
