# Supplementary material for: Psychological factors associated with foot and ankle pain: a mixed methods systematic review
Source: J Foot Ankle Res. 2022 Feb 3;15:10. doi: 10.1186/s13047-021-00506-3 (PMC8812226; doi:10.1186/s13047-021-00506-3)
Supplement: Supplementary file 2 — Additional file 2:. JBI QARI Data Extraction Tool for Qualitative Research_Ceravolo (Data extracted by Frescos). [file 13047_2021_506_MOESM2_ESM.docx]

Table of Contents

[JBI QARI Data Extraction Tool for Qualitative Research_Ceravolo (Data extracted by Frescos) 2](#_Toc88217176)

[JBI QARI Data Extraction Tool for Qualitative Research_Ceravolo (Data extracted by Cotchett) 7](#_Toc88217177)

[JBI QARI Data Extraction Tool for Qualitative Research_Cotchett (Data extracted by Frescos) 12](#_Toc88217178)

[JBI QARI Data Extraction Tool for Qualitative Research_Cotchett (Data extracted by Cotchett) 18](#_Toc88217179)

[JBI QARI Data Extraction Tool for Qualitative Research_Yeowell (Data extracted by Frescos) 28](#_Toc88217180)

[JBI QARI Data Extraction Tool for Qualitative Research_Yeowell (Data extracted by Cotchett) 36](#_Toc88217181)

[JBI QARI Data Extraction Tool for Qualitative Research_Turner (Data extracted by Frescos) 44](#_Toc88217182)

[JBI QARI Data Extraction Tool for Qualitative Research_Turner (Data extracted by Cotchett) 52](#_Toc88217183)

[JBI QARI Data Extraction Tool for Qualitative Research_McAuliffe (Data extracted by Frescos) 58](#_Toc88217184)

[JBI QARI Data Extraction Tool for Qualitative Research_McAuliffe (Data extracted by Cotchett) 65](#_Toc88217185)

## JBI QARI Data Extraction Tool for Qualitative Research_Ceravolo (Data extracted by Frescos)

| **Reviewer: Nikki Frescos** | **Date: 7/12/2020** |
| --- | --- |
| **Author: Ceravolo** | **Year: 2018** |
| **Journal:** Clin J Sport Med | **Record Number** |

**Study Description**

**Methodology**

Interpretive paradigm

Focus group analysed for emergent themes using grounded theory

**Method**

**Interpretive description**

**Phenomena of interest**

Patient’s experience of living with Achilles tendinopathy

**Setting**

On-line survey distributed through email and social media

Focus groups and interviews conducted held at the University of Canberra

**Geographical**

Not indicated but conducted from University of Canberra

**Cultural**

Not indicated

**Participants**

11 participants for the focus group

8 of them had current Achilles tendon pain

Pain location was within 0 to 6 cm from the Achilles insertion,

Age between 18 – 70 yrs old

VISA-AS score (P = 0.427), AQoL-

8D score (P = 0.239), or

pain duration (P = 0.385).

!85 respondents to online survey

**Data analysis**

Thematic analysis for focus group

**Authors conclusions**

The higher-order themes were as follows: (1) adapting lifestyle; (2) living with the condition; (3) changes in mental well-being; (4) conflict with identity; (5) frustration; (6) individual experiences; and (7) changes in social well-being.

Comments

Complete Yes No

| Findings | Illustration from publication (page number) | Evidence | | |
| --- | --- | --- | --- | --- |
|  |  | Unequivocal | Credible | Unsupported |
| *Adapting Lifestyle*  Participants described making changes to their normal lifestyle and physical expectation.  Unable to participate in their normal lifestyle, changes in mood and lost social connections. | “There are things that you can do, whereas I don’t know about  you guys, but when I had the Achilles, it was like being in jail,  that real restriction of your freedom for things that you enjoy  doing.” |  | X  X |  |
| *Living with the condition*  Participants were adapting their lifestyles because they anticipated they would have to live with condition long term.  Some participants reported minimal impact on QoL | “I expect it’s something I’ll be managing forever, by the sounds  of it, from those people who seem to have it.”  “So I just got back to that stage where I’m like—yeah, it’s almost  like accepting you’ve got cancer.”  “An Achilles is not an epic thing where you can’t live a normal life  or anything like that; I can’t say that it overly affected my  quality-of-life.” |  | X |  |
| *Change in Mental Well-Being*  Some participants reported impact on self-esteem, resulting in depression and stress.  Reduced self -esteem was reported as a key contributor to poorer QoL. | “I don’t want to overstate the cranky and anger stuff, but there’s  definitely a general feeling of—it’s almost depression, but not  clinical depression, but you just don’t feel good about yourself  or the world.”  “So I rely on exercise heavily to maintain my mental health. The  endorphins that I get from training are vital to my mental wellbeing.  To not be able to train would be a devastating impact I  think on my mental health.” |  | X  X |  |
| *Conflict with Identity*  Change in fitness levels, impacted on participants’ self-image and self-esteem.  Differences in impact of QoL between participants who reported impact on their self image than those who did not. | “I’d also say that, in some respects, it (running) defines who I  am, and so, if I can’t do that, it’s taking who I am away from me.”  “That’s tough… Well the self-esteem certainly goes down,  because you’re not who you were.” |  | X  x |  |
| *Individual experiences*  Participants previous experiences and beliefs about their condition influenced their  1) coping mechanism and approach.  2) decision on treatment options and perceived prognosis.  There was fear of reoccurrence of the injury which prevented them from activities. | “The attitude in my case, because having 40 years of chronic  back pain off and on, you tend to say absorb it into the  normality of things … Adopt a more practical and stoic  approach to it I think.”  “So they tell you never to stop activity, but I think you really need  to do that with Achilles.”  “There is the fear of it reoccurring … the fear of triggering an  attack prevents you from doing stuff.” |  | X  X | X |
| *Frustration*  Some participants were frustrated in the difficulty in explaining the injury.  Participants reported frustration with being forced into lifestyle adaptations and living with the condition. | “Yeah, I suppose, unless you’ve had it, it’s one of those injuries  that people just wouldn’t have any idea how debilitating it can be.”  “When you don’t see things fixing themselves quickly, in like  a week or 2, and there’s no immediate relief, I think that  starts—you start to get a bit frustrated and annoyed.” |  |  | X  x |
| *Change in Social Well-being*  Participants physical activity is the centre of their social networks.  Participants reported spending less time seeing friends and socializing in groups  Some participants reported it impacted on their family relationships, some stated it had not impact on family life. | “My whole social network is full of runners, so, rather than get  cranky, I’ve stopped going, so, you’re actually withdrawing  from the social networks.”  “My family wasn’t going to go and me stay at home, so, we  never did it and so, we’re actually not with those friends  anymore; so, I think that’s a big impact.” |  | X  X | X |

## JBI QARI Data Extraction Tool for Qualitative Research_Ceravolo (Data extracted by Cotchett)

| **Reviewer: Matthew Cotchett** | **Date: 7/12/2020** |
| --- | --- |
| **Author: Ceravolo** | **Year: 2018** |
| **Journal:** Clin J Sport Med | **Record Number** |

**Study Description**

**Methodology**

Interpretive paradigm

Grounded theory

**Phenomena of interest**

Participant’s experience of living with Achilles tendinopathy

**Setting**

On-line survey

Focus groups and interviews (University of Canberra)

**Geographical**

Not indicated but conducted from University of Canberra

**Cultural**

Not indicated

**Participants**

11 participants for the focus group

The 11 participants who participated in the focus groups were not systematically different to the individuals who completed the survey for VISA-AS score (P 5 0.427), AQoL8D score (P 5 0.239), or pain duration (P 5 0.385). Of the 11 focus group participants, 8 of them had current Achilles tendon pain and 5 had a comorbidity. The comorbidities were Hashimoto disease (n51), low back pain (n51), depression (n51) and high blood pressure (n52)

185 participants responded to the online survey

**Data analysis**

Thematic analysis for focus group

**Authors conclusions**

The higher-order themes were as follows: (1) adapting lifestyle; (2) living with the condition; (3) changes in mental well-being; (4) conflict with identity; (5) frustration; (6) individual experiences; and (7) changes in social well-being.

Comments

Complete Yes No

| Findings | Illustration from publication (page number) | Evidence | | |
| --- | --- | --- | --- | --- |
|  |  | Unequivocal | Credible | Unsupported |
| Adapting lifestyle  Participants noted having to adapt their lifestyle due to their AT which impacted on social connections  Participants were unable to participate in their normal lifestyle, which was associated with lost social connections and changes in mood | “There are things that you can do, whereas I don’t know about you guys, but when I had the Achilles, it was like being in jail, that real restriction of your freedom for things that you enjoy doing”. |  | X |  |
|  |  |  | X |  |
| Living with the condition  Participants described variable experiences related to living with their AT. Some were resigned to living with it. For others did not impact on their quality of life | “I expect it’s something I’ll be managing forever, by the sounds of it, from those people who seem to have it.  “So I just got back to that stage where I’m like—yeah, it’s almost like accepting you’ve got cancer.  “An Achilles is not an epic thing where you can’t live a normal life or anything like that; I can’t say that it overly affected my quality-of-life”. |  | X |  |
| Change in mental wellbeing  Some participants noted the negative impact on their mental health including feeling depressed, stressed and reduced self esteem.  AT limited exercise which negatively impacted on mental health. Exercise relieves stress and increases energy.  Participants reported self-esteem was reduced and was a poor contributor to QoL | “I don’t want to overstate the cranky and anger stuff, but there’s definitely a general feeling of—it’s almost depression, but not clinical depression, but you just don’t feel good about yourself or the world”. |  | x |  |
|  | “So I rely on exercise heavily to maintain my mental health. The endorphins that I get from training are vital to my mental well- being. To not be able to train would be a devastating impact I think on my mental health”. |  | x |  |
|  |  |  |  | X |
| Conflict with identity  Participants who identified as physically fit described a negative impact on self image and self esteem. Quality of life appeared to be more negatively impacted for those whose self image was affected. | I’d also say that, in some respects, it (running) defines who I am, and so, if I can’t do that, it’s taking who I am away from me. That’s tough... Well the self-esteem certainly goes down, because you’re not who you were. |  | X |  |
| Individual experiences  Previous experiences with MSK injuries helped participants cope with AT.  Beliefs about MSk conditions influenced treatment options that they sought and their perceptions of the prognosis | “The attitude in my case, because having 40 years of chronic  back pain off and on, you tend to say absorb it into the  normality of things … Adopt a more practical and stoic  approach to it I think.” |  | x |  |
|  | “So they tell you never to stop activity, but I think you really need  to do that with Achilles.” |  |  | x |
|  | “There is the fear of it reoccurring … the fear of triggering an  attack prevents you from doing stuff.” |  | x |  |
| Individual experiences  Some participants were fearful of re-injury which prevented wanting to exercise | There is the fear of it reoccurring ... the fear of triggering an attack prevents you from doing stuff. |  | X |  |
| Frustration  Some participants described being frustrated because they needed to adapt their lifestyle | “When you don’t see things fixing themselves quickly, in like  a week or 2, and there’s no immediate relief, I think that  starts—you start to get a bit frustrated and annoyed.” |  |  | X |
| Frustration  Some participants were frustrated in their difficulty explaining the injury to others | “Yeah, I suppose, unless you’ve had it, it’s one of those injuries  that people just wouldn’t have any idea how debilitating it can be.” |  |  | X |
| Change in social wellbeing  Participants physical activity is the centre of their social networks  Participants reported spending less time seeing friends and socialising in groups  For some participants there was no impact on social connections  Some participants reported it impacted on their family relationships | My whole social network is full of runners, so, rather than get cranky, I’ve stopped going, so, you’re actually withdrawing from the social networks. |  | X |  |
|  | My family wasn’t going to go and me stay at home, so, we never did it and so, we’re actually not with those friends anymore; so, I think that’s a big impact. |  | x |  |
|  |  |  |  | X |
|  | My family wasn’t going to go and me stay at home, so, we never did it and so, we’re actually not with those friends anymore; so, I think that’s a big impact. |  | X |  |

## JBI QARI Data Extraction Tool for Qualitative Research_Cotchett (Data extracted by Frescos)

**JBI QARI Data Extraction Tool for Qualitative Research**

| **Reviewer** Nikki Frescos | **Date**2/2/2021 |
| --- | --- |
| **Author** Cotchett M et al | **Year** 2020 |
| **Journal** JFAR | **Record Number** |

**Study Description**

Methodology: semi structured interviews

Method

Interpretive description: qualitative descriptive design using the Framework approach

Phenomena of interest: the lived experience and attitudes of people with planter heel pain

Setting: interview

Geographical: metropolitan Melbourne, and the central regional area of the State of Victoria

Cultural: English speaking

Participants: eighteen participants, 12 females 6 males. Age 58.2(6.6), duration of heel pain 15.9(16.3) months.

Data analysis: Framework approach

Authors conclusions: “PHP negatively impacts health-related quality of life. Participants wanted their pain eliminated and reported that their expectations and needs were frequently unmet. Health professionals have an important role to be responsive to the needs of the patient to improve their knowledge and influence pain and behaviour.”

Comments

Complete Yes

| Findings | Illustration from publication (page number) | Evidence | | |
| --- | --- | --- | --- | --- |
|  |  | Unequivocal | Credible | Unsupported |
| ***1.Impact of PHP*** |  |  |  |  |
| Negative impact on physical function. | *“I don’t feel as strong in my whole body. I have a bit more trouble lifting, trouble walking, especially down slopes or downstairs, not so bad going up stairs or up slopes, but activity has certainly been slowed right down”* | X |  |  |
| Reduction of physical activities was associated with psychological impact | *“I’m a mouse on a wheel. I can’t seem to get off. I don’t know what to do. I don’t know how to lose weight without moving, and how do you move without the pain? So yeah, sometimes I’m a bit exasperated by it”* |  | x |  |
| Experience of pain was an obstacle to being socially active, some participants expressed feeling of social isolation. |  |  |  | x |
| Negative social impact: not going on holidays or entertain friends. |  |  |  | x |
| ***2. Perceptions about PHP*** |  |  |  |  |
| Beliefs about the cause | *“Probably I was overdoing it. I increased my walk because I used to do around the three to four kilometres, pushing every now and then. But since I retired, I’ve been going five and at times eight (kilometres), and then walking on both hard surface and the sand”*  *“I just thought it was because I put on a lot of weight during that time”* | X  x |  |  |
| Beliefs about the underlying pathology | *“Well, I really don’t know. I didn’t understand what it was. I knew it had something to do with the plantar fascia”* |  | x |  |
| Beliefs about what the pain meant, a signal of threat or warning sign | *“I feel like pain is my trigger to just try and do something a little bit different”* | x |  |  |
| **3.Coping with PHP** |  |  |  |  |
| Using several different interventions with minimal relief | *“Either I’m not particularly disciplined at doing that (stretching and strengthening) or it just didn’t actually help – maybe I was looking for a quick fix and it didn’t happen quickly enough, and I became a bit frustrated with that”*  *“Well, talk about painful, they were dreadful and then most of my shoes, I couldn’t wear because my instep is too high, and I couldn’t get the foot in the shoe with the orthotics in it”* | x | X |  |
| Differences of opinion in the effectiveness of modifying behaviour | *“I didn’t realise that this would help but I’ve started running and I’ve stopped eating sugar and I don’t think the sugar has a relationship but what it has done is help me lose weight and get healthy”*  *“Maybe I just didn’t give it long enough, but I did have a week of total rest and it didn’t help my foot and my brain nearly went into a massive meltdown”*  *“Go invest in a good pair of shoes firstly. Never walk barefoot or in thongs and even if you get up in the middle of the night, make sure you put a shoe on to walk around”* | X  X  x |  |  |
| Feeling frustrated with lack of consistent and effective treatment approaches | *“But honestly, if someone told me to mix up a special drink ‘cause that’s what was gonna fix it, that’s probably what I’d do as well. So, I probably am trying anything. It’s a bit like spin the wheel and try your luck. I’ll try it all”*  *“I mean there’s so much conflicting information on the internet, trying to put that into context with what doctors told me, what my physio friend at work told me and reading different things on the internet, trying to relate that to my condition, and work it all out” (came from a different theme)* |  | X  x |  |
| ***4.Source of information*** |  |  |  |  |
| Gathering information from various sources to understand and improve PHP was variable | *“I did actually go to the doctor once and described it to him, but – yeah, he just said massage (the foot). The GP didn’t look in the slightest bit interested, really”*  *“A lot of descriptions of very long words that I didn’t really understand … it gave a description about what it was, but actually I didn’t find anything that was really telling me what to do”*  *“Not everything that you read on the internet is possibly correct, so I just dealt with it. I mean, some physicians are not always correct too, but I just felt more secure in actually seeing someone and talking to them face to face rather than reading it off the internet”* | X  X  X |  |  |
| **5. Patient needs** |  |  |  |  |
| Needing better understanding of PHP, and seeking better management | *If I had a better idea, better understanding of what was actually happening with it, I think that would have guided me a lot better in what I was doing about it”*  *“I know that’s a very subjective sort of thing, but to have some idea of – okay, this could take months, it could take three months, it could take six months – just to have some sort of idea, I think, of how long it might take ‘cause I really thought I’d be over it by now”*  *“I want some clear information. Maybe I have seen in some instances for other situations where people have a handout and there are a couple of little photos with brief explanations and maybe the five top suggestions, something to take home”*  *“I mean, there may be no magic bullet here to say we can get rid of it, but if there’s a way to stop the pain, so you can be more active and – yeah, I’m all ears. I’m open to whatever is available”*  *“A lot of the education is being well, “Do this or do that,” but without really explaining what it is that you’re doing and why you’re doing it and what you’re supposed to achieve”*  *“I want videos that show where pain is on the foot, having that pointed out – drawn on a foot, so I can replicate it on mine, all that sort of – if your pain is here, it could be this. If it’s here, it’s probably not this”*  *“I want to see like research-based information. I did find my physio therapist was good at saying, “Oh, this is the latest evidence-based practise for how to help with your hamstring tear.” We didn’t really go to heel descriptions of research. So, I think it should be informed by that and that that should be a clear link”*  *“Pretty much all of the websites that I looked at had the same sort of material just in different words, different diagrams, but not really getting down any deeper than that, and I think I really wanted to understand a bit more about it”*  *“The idea is to gather as much information as possible from various sources regardless of whether they are a podiatrist, doctor, friend, or family, because sometimes you may get the best advice from people who have experienced this sort of problem”* | X  X  X  X  X  X  X  x  x |  |  |
| **6.Advice to others** |  |  |  |  |
| Advising others to seek early diagnosis and professional advice | *“Get a good diagnosis and someone who knows what they’re doing. I suppose looking back, getting a firmer diagnosis at the beginning maybe, trying to get some information that seemed to relate more specifically to your own condition because I found that I was never really quite clear on what advice I was getting really related to what I had because of confusion over what it was”* | x |  |  |

## JBI QARI Data Extraction Tool for Qualitative Research_Cotchett (Data extracted by Cotchett)

| **Reviewer: Matthew Cotchett** | **Date 29/01/2021** |
| --- | --- |
| **Author :Cotchett et al.** | **Year 2020** |
| **Journal : JFAR** | **Record Number** |

**Study Description**

Methodology

A qualitative, interpretive description design – phenomenology

Phenomena of interest: lived experience of people with PHP

Setting: Interview conducted in a University setting

Geographical: Bendigo and Bundoora

Cultural: English speaking

Participants:


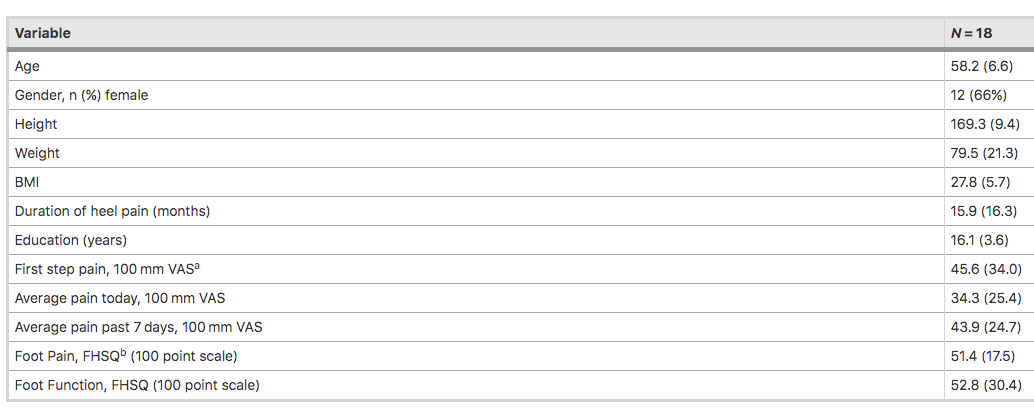


Data analysis: Framework approach

Authors conclusions

The Framework analysis resulted in eight themes with 43 sub-themes (Supplementary file [1](https://jfootankleres.biomedcentral.com/articles/10.1186/s13047-020-0377-3#MOESM1)). The eight themes included ‘perceptions of PHP’, ‘impact on self’, ‘dealing with PHP’, ‘source of information’, ‘patient needs’, ‘patient unmet needs’, ‘advice to others’ and ‘interest in online education’.

The data revealed that PHP negatively impacts health-related quality of life. Participants reported that they wanted their pain eliminated, although their expectations and needs were frequently unmet. Our findings point to the potential importance of providing patient-centred care that considers, and is responsive to, patients’ needs and expectations as well as giving a clear guidance about delivery and content.

Comments

Complete Yes No

X

| Findings | Illustration from publication (page number) | Evidence | | |
| --- | --- | --- | --- | --- |
|  |  | Unequivocal | Credible | Unsupported |
| **Impact of PHP** | | | | |
| Physical | *“I don’t feel as strong in my whole body. I have a bit more trouble lifting, trouble walking, especially down slopes or downstairs, not so bad going up stairs or up slopes, but activity has certainly been slowed right down” (Participant 17).* |  | X |  |
|  |  |  |  |  |
| Emotional  A reduction in physical activity was associated with negative emotions and feelings including sadness, hopelessness and frustration | *I’m a mouse on a wheel. I can’t seem to get off. I don’t know what to do. I don’t know how to lose weight without moving, and how do you move without the pain? So yeah, sometimes I’m a bit exasperated by it” (Participant 15).* |  | X |  |
| Perceptions about PHP | | | | |
| Causal factors  Numerous causes were proposed by participants including being overweight, a change in the level of activity, standing for long periods, walking on hard, soft and uneven surfaces, and walking barefoot | *Probably I was overdoing it. I increased my walk because I used to do around the three to four kilometres, pushing every now and then. But since I retired, I’ve been going five and at times eight (kilometres), and then walking on both hard surface and the sand”* |  |  |  |
|  | *I just thought it was because I put on a lot of weight during that time.* |  |  |  |
| Pathology  Participants reported numerous descriptions of the underlying pathology including plantar fasciitis, bruised heel, broken bone, heel spur and nerve irritation but many were unsure. | *Well, I really don’t know. I didn’t understand what it was. I knew it had something to do with the plantar fascia* |  | x |  |
| Meaning of pain | *Feel like pain is my trigger to just try and do something a little bit different* |  | X |  |
| Coping with PHP | | | |  |
| Participants described a myriad of interventions for PHP including orthomechanical, physical therapies, exercise, pharmacological and strategies to modify their activity. The most common interventions were foot orthoses, exercise, taping and advice regarding footwear, although a few participants combined multiple interventions. Participants’ descriptions of the exercise prescription, which included either stretching or strengthening, were variable. Some participants reported that exercise provided short-term relief, while one participant questioned the effectiveness of exercise | Either I’m not particularly disciplined at doing that (stretching and strengthening) or it just didn’t actually help – maybe I was looking for a quick fix and it didn’t happen quickly enough, and I became a bit frustrated with that” |  | X |  |
| Foot taping was reported to be associated with short term pain relief, although participants reported adverse events such as skin irritation |  |  |  | X |
| Prefabricated foot orthoses had been explored by participants but with mixed success: | *Well, talk about painful, they were dreadful and then most of my shoes, I couldn’t wear because my instep is too high, and I couldn’t get the foot in the shoe with the orthotics in it”*  *“The thing I’ve had the most success with is some orthotic inner soles that are very rigid and seem to hold my foot still. That seems to give me the most support”* |  |  |  |
| Footwear played a key role in alleviating pain with participants highlighting the importance of wearing supportive shoes with a small degree of heel elevation, and caution was expressed when walking in flat unsupportive shoes: | *Go invest in a good pair of shoes firstly. Never walk barefoot or in thongs and even if you get up in the middle of the night, make sure you put a shoe on to walk around”* | X |  |  |
| Tension existed in participants’ responses relating to the role of modifying activity and or rest. While some participants modified their behaviour by limiting or eliminating an activity, others questioned the importance of rest | *I didn’t realise that this would help but I’ve started running and I’ve stopped eating sugar and I don’t think the sugar has a relationship but what it has done is help me lose weight and get healthy”*  *“Maybe I just didn’t give it long enough, but I did have a week of total rest and it didn’t help my foot and my brain nearly went into a massive meltdown”* |  |  |  |
| Overall, a common theme reported by participants was the implementation of a simple trial and error approach |  |  |  | X |
| This was associated with a sense of frustration about the lack of consistency in treatment approach and the absence of treatments known to work for patients: | *But honestly, if someone told me to mix up a special drink ‘cause that’s what was gonna fix it, that’s probably what I’d do as well. So, I probably am trying anything. It’s a bit like spin the wheel and try your luck. I’ll try it all”* |  | X |  |
| Patient needs | | | |  |
| Participants wanted clear explanations of the diagnosis, aetiology, prognosis and treatment options: | *If I had a better idea, better understanding of what was actually happening with it, I think that would have guided me a lot better in what I was doing about it”* |  | X |  |
|  | *“I know that’s a very subjective sort of thing, but to have some idea of – okay, this could take months, it could take three months, it could take six months – just to have some sort of idea, I think, of how long it might take ‘cause I really thought I’d be over it by now* |  | X |  |
| People with PHP wanted face-to-face consultations with a health professional that appeared interested, asked appropriately directed questions, and delivered information in a clear, confident and easy to understand manner |  |  |  | X |
| Online resources were more cautiously viewed, with material being seen on a spectrum from valuable to requiring caution: | *Not everything that you read on the internet is possibly correct, so I just dealt with it. I mean, some physicians are not always correct too, but I just felt more secure in actually seeing someone and talking to them face to face rather than reading it off the internet* | X |  |  |
| Some participants expressed a desire to be given educational resources, such as patient handouts, which provide an outline of the plan recommended by the clinician that can be revisited when suitable for them: | *I want some clear information. Maybe I have seen in some instances for other situations where people have a handout and there are a couple of little photos with brief explanations and maybe the five top suggestions, something to take home* | X |  |  |
| Patient unmet needs | | | |  |
| Participants described unmet expectations regarding explanations of the underlying pathology, causal factors, and descriptions of interventions | *A lot of the education is being well, “Do this or do that,” but without really explaining what it is that you’re doing and why you’re doing it and what you’re supposed to achieve” (Participant 3).* | X |  |  |
|  | *“Here’s half a dozen things that you should try in order of how frequently they assist people with similar problems,” I think. So, like a bit of a format or a plan of what to do and when to move to the next step”* | X |  |  |
| Participants expressed frustration with conflicting information regarding the best approach to manage PHP with a spectrum of messages being delivered by clinicians and resources available onlin | *I mean there’s so much conflicting information on the internet, trying to put that into context with what doctors told me, what my physio friend at work told me and reading different things on the internet, trying to relate that to my condition, and work it all ou* |  | X |  |

##

## JBI QARI Data Extraction Tool for Qualitative Research_Yeowell (Data extracted by Frescos)

| **Reviewer:** Nikki Frescos | **Date 22/3/21** |
| --- | --- |
| **Author:** Yeowell. G et al | **Year** 2021 |
| **Journal:** Physiotherapy | **Record Number** Vol 110 (2021), p 70 -76 |

**Study Description**

**Methodology**

Exploratory qualitative design, using Gadamerian hermeneutic phenomenology

**Method**

Interpretive in depth, one to one, semi-structure interviews. Followed COREQ

**Phenomena of interest**

Explore the lived experiences of people with painful ankle OA.

**Setting**

“in a setting of the participants’ choosing”

**Geographical**

North West England – NHS (seven were recruited from NHS Orthopaedic waiting list)

**Cultural**

Unknown

**Participants**

Nine: Eight participants were males

Mean age 55 years (IQR = 42.5 – 64.5)

Median duration of symptoms 2 years (IQR = 1.5 -10)

**Data analysis**

Thematic analysis

**Authors conclusions**

Findings suggest that those with OA ankle suffer with sever pain, which has a substantial negative impact on person’s physical and mental wellbeing.

Comments

Complete Yes

| Findings | Illustration from publication (page number) | Evidence | | |
| --- | --- | --- | --- | --- |
|  |  | Unequivocal | Credible | Unsupported |
| *1.Signs and Symptoms*  Pain was central issue for participants in terms of type intensity and persistence.  Substantial impact on their life  Participants fearing the pain they might experience if they took part in any physical activity and how fear was preventing them from engaging in this.  Fear of engaging in activities  Physical appearance (swelling) of ankle | “The pain is horrific. It’s just terrible, I wish someone could . . .you know what, I would have it cut off and a false one there if they could. It’s horrendous pain, it’s terrible. It’s driving me round the bend. I’d go for anything to get rid of this pain, I’d try anything now.”  It’s just always there and it just hurts. If it was just one or two blasts of tooth ache then you’d be ok; “ooh that’s not nice”, but when it’s there every time you walk, it just gets you down”  “I can live with the [OA] hip – but I’m not living well with this right ankle. It’s just stopping the living of life, I can’t live my life with it, it’s crazy.”  “I can get away with doing it, it’s afterwards, when I stop, it doesn’t, it’s endless . . . but wow the pain I felt over the following days. It’s like we’d go to the park and I don’t want to risk it. I don’t want to risk it and then it affect me; not moving tomorrow.”  “If I want to play golf I just go out in a buggy [golf buggy to avoid walking], but you do a lot of twisting. And I can see it, even though I’ve got my socks on, I can see it is all swollen and it’s throbbing.”  Anything that’s got rough terrain I don’t want it, I won’t go on it. I might be walking and I hit uneven ground and it gives way.”  “My ankle just doesn’t feel strong. I don’t like walking on cobbles or uneven surfaces as it feels I will go over on my ankle.”  “It’s so swollen, it’s disfigured; it looks quite horrendous.”  “If I want to play golf I just go out in a buggy [golf buggy to avoid walking], but you do a lot of twisting. And I can see it, even though I’ve got my socks on, I can see it is all swollen and it’s throbbing.”  “I have an ankle brace, it’s really good . . . of course in the summer time they’re not great – That’s because it’s hot, and your feet start sweating more. You get sores on your feet with them, so I take them off.” | X  X  X  X  X  X  X | X  X  X |  |
| *2.Impact on participation*  The symptoms experienced impacted on their function and social life | “We tend to go the climbing centre and I’m very limited to where I can go. My friend was into his hiking and we went on loads of walks and I just can’t go.”  “I get no enjoyment out of it [dancing] like I used to . . . and that means I’ve stopped doing that, because I’m not sufficiently ‘macho’ that I will force myself to do it if it hurts.”  “I’m sat in the house, I can’t do nothing. I have no social life. My friends, they all say come and have a pint, but what’s the good in going for a pint when I’m sat there, I can’t move, I can’t go to the bar, I can’t get to the toilet.” | X  X  X |  |  |
| *3. Impact on self-identity and mental wellbeing*  Participants felt that it impacted their self-identity and self-worth.  Participant had feelings of depression and affected their mental wellbeing. | “I was like the leader, and them the handbrakes because they were slower than me; now I’m the handbrake.”  “I’m just useless, just because of a daft ankle. It’s unbelievable that isn’t it. It makes me feel as if I’m good for nothing, I might as well just turn it in, you know, just go for a couple of tablets and I’ll call it a day. Just a waste of time. I’m good for nothing at the minute. I feel like crying. It’s horrible. Everyday of my life; it gets a bit upsetting. You just wanna give in, in the end, you get sick of it.”  “I got quite depressed with it all. I didn’t realise that there was such an adverse effect that the pain  grinds you down and gives you that low self-esteem and no self-confidence. You can see other people your age doing things but you’re not able to. It wears you down mentally and makes you very depressed at times. What you don’t realise is it’s not just physical, it very much you mentally.”  “I got quite depressed with it all. I didn’t realise that there was such an adverse effect that the pain grinds you down and gives you that low self-esteem and no self-confidence. You can see other people your age doing things but you’re not able to. It wears you down mentally and makes you very depressed at times. What you don’t realise is it’s not just physical, it very much affects you mentally.” | X  X |  |  |
| *4.Views on non- surgical management*  Participants found non- surgical interventions helpful  Participants found non- surgical interventions not helpful  Participants wore high ankle boots to provide ankle support but not aesthetic option.  Participants found that their condition was not taken seriously which impacted their mental and physical well- being and quality of life. | “It was gentle exercises, which helped the stiffness in the joint. Doing any kind of mild exercise was unbelievable and the manipulation stuff helped because it kept the joints from freezing up. So, they definitely helped.”  “I have an ankle brace, it’s really good . . . “  “The physio gave me some exercises which, quite frankly, didn’t really help . . . it was just movement exercises and strengthening – the idea being to strengthen the joint.”  “And I wear boots just pass the ankle and I strap them up around me ankle. So it looks stupid when you wear boots”  “They x-rayed it, and they said it’s not too bad. They said you can see it, but it’s not bad . . . but I can barely walk on it [in the mornings] until it wakes itself up a bit.”  “In some sense, it seems that it’s not being treated seriously, and if I physically couldn’t walk, I’d be referred to people. Actually, it matters. Long before people are physically incapacitated, because it’s affecting my mental health, it’s affecting my physical health, but it’s not extreme enough with the level of funding the NHS has at the moment for it to be treated seriously. With ankle pain, the pain doesn’t have to be that bad to have a massive impact on your quality of life.” | X  X  X  x  X | X |  |
|  |  |  |  |  |
|  |  |  |  |  |
|  |  |  |  |  |

## JBI QARI Data Extraction Tool for Qualitative Research_Yeowell (Data extracted by Cotchett)

| **Reviewer: Matthew Cotchett** | **Date** |
| --- | --- |
| **Author Yeowell et al** | **Year2021** |
| **Journal** | **Record Number** |

**Study Description**

*Methodology*

Interpretive description

Method: Semi-structured interviews

Phenomena of interest: To explore the experiences of people living with painful OA ankle and their views about the non-surgical management of this condition.

Setting: an orthopaedic clinic at a UK hospital and general population

Geographical: UK

Cultural

Participants: 9 participants (8=M; 1=F); median age =55 years; median duration of symptoms = 2 years

Data analysis: Thematic analysis

Authors conclusions:

Four themes were identified:

• Signs and symptoms
• Impact on participation
• Impact on self-identity & mental wellbeing • Viewsonnon-surgicalmanagement

Our findings suggest that those with OA ankle suffer with severe pain, which has a substantial negative impact on a person’s physical and mental wellbeing.

Comments

Complete Yes No

| Findings | Illustration from publication (page number) | Evidence | | |
| --- | --- | --- | --- | --- |
|  |  | Unequivocal | Credible | Unsupported |
| Signs and symptoms | | | | |
| Two main symptoms (pain instability) and one sign (swelling) |  |  | X |  |
| Pain is the central issue | The pain is horrific. It’s just terrible, I wish someone could . . . you know what, I would have it cut off and a false one there if they could. It’s horrendous pain, it’s terrible. It’s driving me round the bend. I’d go for anything to get rid of this pain, I’d try anything now. |  |  |  |
| Swelling is a key feature | It’s so swollen, it’s disfigured; it looks quite horrendous. |  | X |  |
|  | If I want to play golf I just go out in a buggy [golf buggy to avoid walking], but you do a lot of twisting. And I can see it, even though I’ve got my socks on, I can see it is all swollen and it’s throbbing. |  | X |  |
| Negative impact of OA | I can live with the [OA] hip – but I’m not living well with this right ankle. It’s just stopping the living of life, I can’t live my life with it, it’s crazy. |  |  |  |
| Unrelenting nature of the pain | It’s just always there and it just hurts. If it was just one or two blasts of tooth ache then you’d be ok; “ooh that’s not nice”, but when it’s there every time you walk, it just gets you down. | X |  |  |
| Fear of pain | I can get away with doing it, it’s afterwards, when I stop, it doesn’t, it’s endless... but wow the pain I felt over the following days. It’s like we’d go to the park and I don’t want to risk it. I don’t want to risk it and then it affect me; not moving tomorrow. |  |  |  |
| Impact of pain on social activities |  |  |  | X |
| Uneven surfaces impact on stability | My ankle just doesn’t feel strong. I don’t like walking on cobbles or uneven surfaces as it feels I will go over on my ankle.  Anything that’s got rough terrain I don’t want it, I won’t go on it. I might be walking and I hit uneven ground and it gives way. |  | X |  |
| Impact on participation | | | | |
| Negative impact on function and social life | We tend to go the climbing centre and I’m very limited to where I can go. My friend was into his hiking and we went on loads of walks and I just can’t go.  I get no enjoyment out of it [dancing] like I used to . . . and that means I’ve stopped doing that, because I’m not sufficiently ‘macho’ that I will force myself to do it if it hurts. | X |  |  |
| Impact on social life |  |  |  | X |
| Impact on QoL | I’m sat in the house, I can’t do nothing. I have no social life. My friends, they all say come and have a pint, but what’s the good in going for a pint when I’m sat there, I can’t move, I can’t go to the bar, I can’t get to the toilet. | X |  |  |
| Impact on self-identity & mental wellbeing | | | | |
| Participants felt that they had experienced a change in their self-identity and perceived this as a loss of self-worth | I was like the leader, and them the handbrakes because they were slower than me; now I’m the handbrake.  I’m just useless, just because of a daft ankle. It’s unbelievable that isn’t it. It makes me feel as if I’m good for nothing, I might as well just turn it in, you know, just go for a couple of tablets and I’ll call it a day. Just a waste of time. I’m good for  nothing at the minute. I feel like crying. It’s horrible. Every day of my life; it gets a bit upsetting. You just wanna give in, in the end, you get sick of it. |  | X |  |
| Negative impact on mood | I got quite depressed with it all. I didn’t realise that there was such an adverse effect that the pain grinds you down and gives you that low self-esteem and no self-confidence. You can see other people your age doing things but you’re not able to. It wears you down mentally and makes you very depressed at times. What you don’t realise is it’s not just physical, it very much affects you mentally. | X |  |  |
| Mixed response to non-surgical interventions – some described a benefit but short lived | The physio gave me some exercises which, quite frankly, didn’t really help . . . it was just movement exercises and strengthening – the idea being to strengthen the joint.  It was gentle exercises, which helped the stiffness in the joint. Doing any kind of mild exercise was unbelievable and the manipulation stuff helped because it kept the joints from freezing up. So, they definitely helped. |  |  |  |
| Participants who used external ankle supports and orthoses found these to be beneficial. However, some participants highlighted the difficulty of donning the support due to swelling in the ankle, and for many there were issues of tolerance and acceptability which impacted their benefit. | I have an ankle brace, it’s really good . . . of course in the summer time they’re not great – That’s because it’s hot, and your feet start sweating more. You get sores on your feet with them, so I take them off. |  | X |  |
| Issues with acceptability of wearing high cut shoes (social acceptability) | And I wear boots just pass the ankle and I strap them up around me ankle. So it looks stupid when you wear shorts. | X |  |  |
| Issues with acceptability for one female participant |  |  |  | X |
| Signs and symptoms not taken seriously in the early stages of disease and the impact this had on mental health | They x-rayed it, and they said it’s not too bad. They said you can see it, but it’s not bad ... but I can barely walk on it [in the mornings] until it wakes itself up a bit. |  | X |  |
|  | In some sense, it seems that it’s not being treated seriously, and if I physically couldn’t walk, I’d be referred to peo- ple. Actually, it matters. Long before people are physically incapacitated, because it’s affecting my mental health, it’s affecting my physical health, but it’s not extreme enough with the level of funding the NHS has at the moment for it to be treated seriously. With ankle pain, the pain doesn’t have to be that bad to have a massive impact on your quality of life. | X |  |  |

## JBI QARI Data Extraction Tool for Qualitative Research_Turner (Data extracted by Frescos)

**JBI QARI Data Extraction Tool for Qualitative Research**

| **Reviewer: NF** | **Date: 9/12/2020** |
| --- | --- |
| **Author: Turner** | **Year: 2020** |
| **Journal: PLoS ONE** | **Record Number** |

**Study Description**

**Methodology**

A qualitative, interpretive description design

**Method**

Semi-structured interview

Convenience sample

**Phenomena of interest**

The lived experiences of individuals with AT.

**Setting**

Telephone interviews

**Geographical**

Melbourne Australia

**Cultural**

English speaking background

**Participants**

15 participants (8 male and 7 female) with AT. Age range 26 -72yo (µ=45.2); either unilateral or bilateral AT; location of symptoms insertional (11), mid portion(3); symptom duration 4 – 96 months (µ=8), runners(10) and non runners(5)

Exclusion: any concurrent injuries to foot, ankle and/or hip on the same side of the AT, current low back pain and/or history of inflammatory arthropathy

**Data analysis**

Thematic analysis

**Authors conclusions**

Results of the current study add to an emerging body of evidence highlighting the substantial fears, frustrations and impact on quality of life and daily functioning in individuals with tendinopathy. Results of this study emphasize the need to address psychosocial factors in AT and include psychological factors as a core health-related domain for the assessment and treatment of tendinopathy. Such insights may have profound implications for informing appropriate clinical practice and underscores the potential for a biopsychosocial approach in the management of AT.

**Comments**

Complete Yes No

| Findings | Illustration from publication (page number) | Evidence | | |
| --- | --- | --- | --- | --- |
|  |  | Unequivocal | Credible | Unsupported |
| *Beliefs and perceptions*  *surrounding AT*  **Beliefs surrounding causation**  Nearly all participants believed that over training/or overuse was a primary cause of their AT and  lack of time for recovery.  Some participants believed that the lack of overall fitness was the key cause for the development of AT.  **Perceptions regarding non-resolution in AT**  There was confusion or lack of understanding as to why their condition had not resolved.  **Frustration with HCP**  Some participants expressed their frustration and dissatisfaction with HCPs. This was due to confusion with the explanation of the condition and treatment required and varying opinions. There was also a sense that the HCPs were not listening to their questions.  **Maladaptive beliefs and avoidance behaviours**  Participants reported modifying or ceasing physical activities due to the fear of further injury, rupture of the tendon or pain. | “So, I feel that I probably over trained. Not so much leading up to the run, it was more I didn't  recover and allow myself time to recover afterwards and I just pushed it a little bit too far.”  “I just assume that I've become slightly unfit and that I always had tight muscles in my legs  and it's kind of a consequence of decades of not really exercising.”  “I don't actually know what's going on. When I feel the pain, I mean I feel it in the base of my  Achilles, but I don't know what's going on.”  “Sometimes when you talk to your doctor, or the specialist, it's very limited time, and they  don't have the time to explain it properly, and they speak in technical terms. I thought the physio  spent a bit more time with you to talk to you about it.”  “Everyone has their sort of different opinions, so it's not, I don't know, not always consistent  information at the same time, as well.”  “….I've got that kind of doubt niggling in the back of my mind about it.  That I need to protect it, rather than let it get too bad. So I'm not someone who would take it  that far to the edge. I think that's probably more of it, it just hinders me from going further or  harder, or any of those things really.”  “I'm quite certain that if I played table tennis, it would definitely be worse. I'm not even  attempting that, because I'm just scared that I might rupture a tendon.”  “If I'm overtraining or something, I don't really know, perhaps the rubbing together of the tendons  causing mini fractures?” |  | X  X  x  x  X  X  X |  |
| *The biopsychosocial impact*  *of AT*  All participants reported that the injury **impacted on their daily routines** but did not have an impact on their work.  Nearly all participants reported that the AT **impacted on their running**, either having reduced or completely stopped running  **Psychological impact** was expressed by several participants in their frustration or annoyance of the condition and its limitation on their activities.  The loss of identify was for some participants more significant than inability to run at their previous levels | “I think it restricts me in a lot of things that I would be able to do. I don't think I can go out  and kick the footy with my son, or. . . You know, I manage to. . . in pain, to go for a walk with  the dog in the evening, if you know what I mean?”  “It just means altering, I guess, my training regime that I'm used to, to kind of fit in with the  injury. So, when there's the running aspect in that, I just don't even bother trying anymore.”  “Well, I think it's just like there's things that I enjoy doing and if I can't do them, now I get a  bit frustrated and it's part of what makes me happy and makes me satisfied with things.”  “... You know, you do a nice, long run and you feel quite  good after it. I'm not having that experience. I think that satisfaction, the challenge, and all  that sort of stuff, I'm just not being able to do and expose myself to and I kind of struggle to  find that in other modes when I'm not running.” | X  x | x | X |
| *Individuals’ experiences with the management*  *process in AT*  Participants stated they were **motivated to seek** treatment for their pain and fear of disability and also further deterioration of both the condition and physical activity.  Positive **experience with passive** treatment such as massage, ultrasound and dry needling) were preferred by the participants and stated as most effective.  Participants **experience with active treatment** was variable. Strength training was believed to be most effective treatment by several participants  Some participants reported difficulty in adhering to the prescribed exercises. And therefore blamed themselves for the lack of improvement.  There was a feeling of lack of control over their condition.  **Motivational barriers** were described for the slow progression of rehabilitation  (not supported) | “And there are still things I want to do in the future, like with running and more marathons,…... So, I don't have a choice but to keep it strong  and keep doing those exercises.”  “So, acupuncture tends to. . . I respond really well to that and pretty quickly as well. Obviously,  massage, anything to loosen up my calf, really. So, massage work or acupuncture on my  calves”  “I'm the only one to blame for it being the way it is. Yeah, I mean it's certainly in my control. I  can't blame anyone else for it being the way it is and I've. . . It's my decision to do or not do my exercises and things like that. I wish there was a magic pill that I could take to resolve it.”  “I failed to go to  the gym this morning, because I was feeling fed up with myself and so I'm not  really in control of any of these things.” *(x credible)* |  | x  X  X  X | X  X |
| *Future prognosis and outlook in*  *individuals with AT*  Both **positive and negative prognostic** outlooks were presented. Most participants expressed optimism with their condition and identified the need for compliance to treatment.  Those who expressed poor prognosis stated chronicity, pathology and genetic disposition as reasons.  Both positive and negative self- efficacy in the ability to control their condition were cited by participants. | “…..I see  myself better and I think of one thing I’ve progressed with is in learning ways to ongoingly  [sic] prevent this sort of injury.”  “It can be relieved, but there's nothing you can do about flat feet. I'm born like that. That's  how life is. I'm not gonna [sic] play tennis again. I'm not sure that I'll be able to play table tennis.”  “It's something that's not going to go away, and if I don't keep up doing the exercises, it's just  going to weaken. I'm always mindful to keep it strong and keep it going well, I guess.”  “I no longer feel like I'm in control of it now.”  "Yeah, I kind of almost resigned myself that it's going to be a long process.” (unsupported) | x | X  X  x | X |
|  |  |  |  |  |
|  |  |  |  |  |
|  |  |  |  |  |

## JBI QARI Data Extraction Tool for Qualitative Research_Turner (Data extracted by Cotchett)

| **Reviewer: Matthew Cotchett** | **Date 18/11/20** |
| --- | --- |
| **Author :Turner 2020** | **Year 2002** |
| **Journal: Plos One** | **Record Number A1** |

**Study Description**

**Methodology**

A qualitative, interpretive description design - phenomenology

**Phenomena of interest**

The lived experience of people with AT

**Setting**

Running clubs, private practice with MSk focus

**Geographical**

Melbourne, Victoria

**Cultural**

English speaking background

**Participants**

Age 26 – 72; Equal M/F ratio (8 male/7 female); AT > 3/12 months duration; mix of runners and non-runners; insertional and midportion AT. Excluded non-English speaking participants

**Data analysis**

Thematic analysis according to Braun and Clarke.

**Authors conclusions**

Four main themes were identified from the data: 1) beliefs and perceptions surrounding AT: *“If I’m over training or something*, *I don’t really know”*, 2) the biopsychosocial impact of AT: “*I think it restricts me in a lot of things that I would be able to do”*, 3) individuals’ experiences with the management process: “*You want it to happen now*. *You’re doing all this stuff and it’s just very slow progress”*, and 4) future prognosis and outlook in individuals with AT: “*I see myself better”*.

Comments

Complete: Yes  No

| **Findings** | **Illustration from publication (page number)** | **Evidence** | | |
| --- | --- | --- | --- | --- |
|  |  | **Unequivocal** | **Credible** | **Unsupported** |
| **Beliefs and perceptions surrounding AT** | | | | |
| Beliefs surrounding causation  Nearly all participants noted that over training was probably the cause of their AT  Overall fitness might be a contributing factor | *“So I feel that I probably over trained. Not so much leading up to the run, it was more I didn’t recover and allow myself time to recover afterwards and I just pushed it a little bit too far”* | X |  |  |
|  | *I just assume that I've become slightly unfit and that I always had tight muscles in my legs and it's kind of a consequence of decades of not really exercising* |  |  |  |
| Perceptions regarding non-resolution in AT  Several participants described having lack of knowledge or being confused as to why their condition had not resolved | *“I don't actually know what's going on*. *When I feel the pain*, *I mean I feel it in the base of my*  *Achilles*, *but I don't know what's going on*.*” C*  *I  feel like I should know more*, *but I don't* |  | X |  |
|  |  |  |  | X |
| Frustration with healthcare providers  Frustrations and/or dissatisfaction with healthcare providers and the education they received was a common theme and was reported by sev- eral participants  Confusion from conflicting information | *“Sometimes when you talk to your doctor*, *or the specialist*, *it's very limited time*, *and they don't have the time to explain it properly*, *and they speak in technical terms*. *I thought the physio spent a bit more time with you to talk to you about it*.*” C* |  | X |  |
|  | *Everyone has their sort of different opinions*, *so it's not*, *I don't know*, *not always consistent information at the same time*, *as well*. |  | X |  |
| Maladaptive beliefs and avoidance behaviours  Nearly all participants reported having significantly reduced or completely ceased certain physical activities due to fear of further injury, damage, and/or pain. | *“I often just pull out earlier then*. . . *I never let it get that bad*, *if you know what I mean*? *I don't really go in as hard*. *I've got that kind of doubt niggling in the back of my mind about it*. *That I need to protect it*, *rather than let it get too bad*. *So I'm not someone who would take it that far to the edge*. *I think that's probably more of it*, *it just hinders me from going further or harder*, *or any of those things really*.*” C* |  | X |  |
| Several participants (7/15) expressed fear of tendon rupture as the reason they had stopped and/or avoided certain physical activities and hobbies.  A few participants (4/15) expressed perceptions of vivid pathological damage to their tendon when asked why they thought they had not gotten better initially. | *“I'm quite certain that if I played table tennis, it would definitely be worse. I'm not even attempting that, because I'm just scared that I might rupture a tendon.” (P14)* | X |  |  |
|  | *“If I'm overtraining or something, I don't really know, perhaps the rubbing together of the tendons causing mini fractures?”* |  | X |  |
| **The biopsychosocial impact of AT** | | | | |
| Impact on daily routine  All participants reported that their daily routines and activities were affected by their AT. | *I think it restricts me in a lot of things that I would be able to do*. *I don't think I can go out and kick the footy with my son*, *or*... *You know*, *I manage to*... *in pain*, *to go for a walk with the dog in the evening*, *if you know what I mean*?*”* | X |  |  |
| The majority of participants (10/15) had sedentary jobs and thus reported having pain while at work, but not reported to be a hindrance to work productivity. | *Not a real impact on my work*. *I guess I've got a reasonably sedentary job* |  |  |  |
| Impact on running  Nearly all the participants reported having reduced or completely stopped running due to their AT. | *“It just means altering*, *I guess*, *my training regime that I'm used to*, *to kind of fit in with the injury*. *So*, *when there's the running aspect in that*, *I just don't even bother trying anymore”*. | X |  |  |
| Psychological impact  A majority of participants described frustration and/or annoyance with their condition and its limitations on activity. | *“It's disappointing and it's pretty frustrating, really, because it feels like it's something that will never go away, but yeah, it's just very frustrating is probably the biggest thing, really.”* |  | X |  |
|  | *“Well*, *I think it's just like there's things that I enjoy doing and if I can't do them*, *now I get a bit frustrated and it's part of what makes me happy and makes me satisfied with things*. *Yeah*, *I think it’s part of those basic sort of*. . . *You know*, *you do a nice*, *long run and you feel quite good after it*. *I'm not having that experience*. *I think that satisfaction*, *the challenge*, *and all that sort of stuff*, *I'm just not being able to do and expose myself to and I kind of struggle to find that in other modes when I'm not running*.*”* |  | X |  |
| **Individual’s experiences with the management process** | | | | |
| Motivations to seek treatment  A majority of participants were motivated to seek treatment by their pain and fear of disability, worsening condition, or further loss of physical activity. | *“And there are still things I want to do in the future*, *like with running and more marathons*, *and even doing some ultras and stuff like that*. *So*, *I don't have a choice but to keep it strong and keep doing those exercises”.* | X |  |  |
| Experience with passive treatment  Nearly all of the participants were prescribed or sought passive treatment for management of their AT. | *So*, *acupuncture tends to*. . . *I respond really well to that and pretty quickly as well*. *Obviously*, *massage*, *anything to loosen up my calf*, *really*. *So*, *massage work or acupuncture on my calves*. |  | *X* |  |
|  | *I've had a lot of massage over the years*. *I've had dry needling in my calves because my calves and hamstrings are tight*, *and this may be contributing to my symptoms*.*”* | X |  |  |
| Experience with active treatment  Several of the participants believed strength training was the most effective treatment in AT | *I'm not sure that the exercise alone*, *without the shock wave*, *would've been effective*. |  |  | X |
| Nearly all of the participants (13/15) were prescribed some form of strength training by HCPs. The most frequently reported type of prescribed strength training was body weight calf raises/heel lifts. Dosages were quite variable and nearly all participants (13/15) reported difficulty with maintaining adherence with their prescribed exercises. A majority of participants (10/15) blamed themselves for the cause of their AT. | *I failed to really maintain it properly*. *I should have done more of those exercises prescribed”* |  |  | X |
|  | *'m the only one to blame for it being the way it is. Yeah, I mean it's certainly in my control. I can't blame anyone else for it being the way it is and I've… It's my decision to do or not do my exercises and things like that. I wish there was a magic pill that I could take to resolve it.”* |  | *X* |  |
| Motivational barriers  Common barriers noted were feelings of lack of control over their condition, perceptions that rehabilitation exercise was tedious and not enjoyable, and frustration with the slow progress of rehabilitation. | *I failed to go to the gym this morning, because I was feeling fed up with myself and so I'm not really in control of any of these things.”* |  | *X (lack of control only)* |  |
| **Future prognosis and outlook** | | | | |
| Positive prognostic outlook  The majority of participants expressed optimism regarding the future of their condition and return to prior levels of activity | *“I see myself better*. *I think I feel like I'm very much in the last stages of this problem*. *I see myself better and I think of one thing I’ve progressed with is in learning ways to ongoingly [sic] prevent this sort of injury”*. | X |  |  |
| **Negative prognostic outlook**  Several participants (5/15) expressed a negative outlook on their future prognosis and ability to return to prior levels of activity | *It can be relieved, but there's nothing you can do about flat feet. I'm born like that. That's how life is. I'm not gonna [sic] play tennis again. I'm not sure that I'll be able to play table tennis.”* |  | *X* |  |
| **Positive self-efficacy**  A majority of participants (8/15) reflected confidence in their ability to exert control over their condition. | *“It's something that's not going to go away*, *and if I don't keep up doing the exercises*, *it's just going to weaken*. *I'm always mindful to keep it strong and keep it going well*, *I guess*. |  | *X* |  |
| **Negative self-efficacy**  Several participants (7/15) reflected a lack of confidence in their ability to exert control over their condition. | *“Yeah*, *I kind of almost resigned myself that it's going to be a long process*.*”* |  |  | X |

Extraction of findings complete: Yes  No

## JBI QARI Data Extraction Tool for Qualitative Research_McAuliffe (Data extracted by Frescos)

**JBI QARI Data Extraction Tool for Qualitative Research**

| **Reviewer NF** | **Date 8/12/2020** |
| --- | --- |
| **Author: McAuliffe** | **Year 2017** |
| **Journal: Musculoskeletal and Practice** | **Record Number** |

**Study Description**

**Methodology**

A qualitative descriptive Interpretative paradigm

**Method**

Semi-structured telephone interviews

**Phenomena of interest**

The perceptions and experiences of people with Achilles Tendinopathy

**Setting**

Telephone interview

**Geographical**

Limerick, Ireland

**Cultural**

English speaking background

**Participants**

Five males and 3 females (although Table 1 shows 6 males and 2 females); age range 33 -51yo; 3x bilateral AT and 5 x unilateral AT, duration of AT range 7 – 96 months; 5 runners and 3 non-runners

**Data analysis**

Thematic analysis

**Authors conclusions**

Persistent AT is associated with a significant psychosocial burden, particularly in terms of participation in daily life and valued activities. There is considerable confusion and frustration in regard to the management of AT and its course. Better understanding of the personal experiences of AT may enhance management of persistent AT and facilitate individuals with AT complying with evidence-based approaches including exercise and pain reconceptualization.

**Comments**

**Complete**  Yes No

| Findings | Illustration from publication (page number) | Evidence | | |
| --- | --- | --- | --- | --- |
|  |  | Unequivocal | Credible | Unsupported |
| *Pain as a feature of everyday life*  **Pain and daily activities**  Participants stated that the pain associated with AT substantially disrupted and changed their ability to perform and engage in daily activities.  The physical disruption varied based on participants symptoms, however morning pain was more common.  Some participants reported disruption at work, depending the demands of work.  Participants described a **loss of self** it affected their personal activities, such as giving up recreational activities (running), impacting on their social life and a sense of personal achievement and sense of self. | “But in the morning times in particular, very sore in the mornings.  You're literally hobbling around the place until you get  moving as such.”  “Aww, it's just when you have to go anywhere, walk around the  office, walking to go get a coffee. You get up; you've forgotten  about it sitting down … once you get up it's like ouch. Then  you're almost limping everywhere. It's noticeable enough in that  people would say are you all right, what are you limping for”.  “Frustration, needing….Wanting to run. Seeing my peers going  to races, winning races or getting PBs. Progressing … and I'm  stuck here. That has been horrendous I have to say. Now, I know  there are worse things in life that can happen. But it's been  horrible.”  “I get frustrated with myself, you know like feels there nothing  at the end of the tunnel you know like. Like I'm not saying me  life is around the running but Like I was an alcoholic and I  haven't had in drink twelve and a half year ago. And i took up  running and it's me life since, you know like.” | X | X  X  X  X | X (loss of self)  X (sense of self) |
| *Participant’s experience with the management process*  Participants outlined their **frustration with the treatment process** and their lack of confidence in the health care practitioners who were treating them  Participants described their **experiences with the treatment process.**  They believed that forceful hands on treatment was required for the treatment to be of benefit.  Also highlighted the role of exercise interventions in the management of AT,  Some participants discussed their lack of confidence and disbelief in the exercise as an intervention | “For me there were a lot of grey areas. Everybody was giving me,  like telling me different things. So like nobody knew 100%  everything about Achilles and it was varying, you know people  opinions were varying.”  “He wore a kind of a knuckle duster and really rubbed them  hard. And I think that's the only thing that got rid of the morning  stiffness.”  “but I know there are some exercises you should be doing and  things like but that. I probably haven't done as much strength  work. I don't go to the gym. I don't do any strength work or  anything like that.”  “There was a therapy that was recommended to me maybe at  the start of the year, it was called heel drops or painful heel  drops? They're on the edge of the steps and you basically flex,  you basically flex down and flex up…Was it 10 minutes a night  every night for 12 weeks but in my head that was crazy.” | X | X  x | x |
| *Identifying with and self-managing AT*  Participants had **beliefs surrounding the causation** of AT and outlined a range of factors they felt were associated with the development of AT.  There was also confusion in relation to the cause.  Rest was considered as a self-management strategy Rest would ease the pain and help with healing. This approach was based on the participants belief or on advice of others (HCP or non HCP)  Some participants did not believe in rest as a management approach and expressed a desire to continue running. | “Obviously there's some weakness there i suppose. Or some, or  is it down to running style or something. Footwear yeah”.  “I don't know. It's a question people often ask. Definitely people  would say to you it's an overuse. And well I'm not doing enough  for it to be overuse. That's what wrecks me.”  “I possibly didn't rest it enough. That's the only assumption I can  come up with that I didn't rest it enough Yeah. As in I've stopped  running altogether in the hope that the rest will allow it to  recover. The problem is its back to the whole thing of not  knowing is that the problem.”  “I've gone to a lot of physios and they tell me its an overuse  injury but I don't think it is ‘cause I've stopped. People say stop  and don't do anything for 6 or 8 weeks but if I did stop and went  back even for a jog a couple of miles after not doing anything for  a period of time id still be in pain like it never stopped.” |  | X  X  X | x |
| *Looking to the future*  Participants **fear of future prognosis** was expressed by their concerns and fears and uncertainty surrounding potential future damage to the Achilles tendon. They feared that continued to exercise or did not address their AT it may lead to greater disability or risk of rupturing their Achilles tendon.  The majority of participants **desired to run again**, to return to previous running levels or re-engaging in running without pain | “Yeah it could like. It probably … I'd be thinking that like. By  running on it as it is, i think it would. It might get to the stage  where it will rupture … If I don't get it fixed like.”  “Being able to go to a class and not have pain like after a class.  Being able to touch my heel without it being painful is a big  thing. That would be it really, not to have pain really.”  “Being able to go to a class and not have pain like after a class.  Being able to touch my heel without it being painful is a big  thing. That would be it really, not to have pain really.” | x | x |  |
|  |  |  |  |  |
|  |  |  |  |  |
|  |  |  |  |  |

## JBI QARI Data Extraction Tool for Qualitative Research_McAuliffe (Data extracted by Cotchett)

| **Reviewer Matthew Cotchett** | **Date 19/11/20** |
| --- | --- |
| **Author McAuliffe** | **Year 2017** |
| **Journal: Musculoskeletal science practice** | **Record Number A2** |

**Study Description**

**Methodology**

A qualitative, interpretive description design - phenomenology

**Phenomena of interest**

The lived experience of people with AT prior to an intervention study

**Setting**

Telephone interviews

**Geographical**

Limerick, Ireland

**Cultural**

English speaking background

**Participants**

Age 33-51; M/F ratio (6 male/2 female); AT > 3/12 months duration; mix of runners and non-runners; 7 x midportion AT; 1 x Insertional; Excluded non-English speaking participants

**Data analysis**

Thematic analysis.

**Authors conclusions**

Four main themes were identified from the data: (i) pain as a feature of everyday life; (ii) participant's experience with the management process; (iii) identifying with and self-managing AT, and (iv) looking to the future. Table 2 presents the categories constituting each theme.

“This study suggests that persistent AT is associated with a significant psychosocial impact, particularly in terms of participation in daily life and valued activities. Better understanding the experiences and personal impacts of AT may enhance management of this persistent disorder, and facilitate individuals with AT complying with evidence-based approaches including exercise and pain reconceptualization.

Comments

Complete Yes No

| **Findings** | **Illustration from publication (page number)** | **Evidence** | | |
| --- | --- | --- | --- | --- |
|  |  | **Unequivocal** | **Credible** | **Unsupported** |
| **Pain as a feature of everyday life** | | | | |
| Pain and daily activities  Many participants stated that the pain associated with AT resulted in a substantial highlighted their experience of pain and the ability to perform daily activities | “The morning pain oh its constant. I'd say I haven't had a morning since the start of the year where coming down the stairs i haven't been very tentative about I”t. |  | X |  |
| Pain and daily activities  Symptoms had an impact for some individuals depending on the job | “Aww, it's just when you have to go anywhere, walk around the office, walking to go get a coffee. You get up; you've forgotten about it sitting down ... once you get up it's like ouch. Then you're almost limping everywhere. It's noticeable enough in that people would say are you all right, what are you limping for |  | X |  |
| **Loss of self** | | | | |
| Participants noted a negative effect on the ability to undertake activities that are enjoyable and contribute to their social life and sense to self | Frustration, needing .... Wanting to run. Seeing my peers going to races, winning races or getting PBs. Progressing ... and I'm stuck here. That has been horrendous I have to say. Now, I know there are worse things in life that can happen. But it's been horrible |  | X linked to first half of finding |  |
|  | I get frustrated with myself, you know like feels there nothing at the end of the tunnel you know like. Like I’m not saying me life is around running but like I was an alcoholic and I haven’t had a drink twelve and a half year ago. And I took up running and it’s me life since you know like. |  | X X linked to second half of finding |  |
| **Participants experience with the management process** | | | | |
| Frustration with the treatment process  Participants noted frustration with the treatment process and the uncertainty of professional opinion | “For me there were a lot of grey areas. Everybody was giving me, like telling me different things. So like nobody knew 100% everything about Achilles and it was varying, you know people opinions were varying”. |  | X |  |
| Experience with the treatment process  Participants expressed the belief that forceful passive treatments were required | “He wore a kind of a knuckle duster and really rubbed them hard. And I think that's the only thing that got rid of the morning stiffness” |  |  | X |
| Experience with the treatment process  Participants noted the expectation of exercise  but were either non-adherent or did not find the principle plausible  …. or did not find the principle plausible | “But I know there are some exercises you should be doing and things like that but that. I probably haven’t done as much strength work. I don’t go to the gym. I don’t do any strength work or anything like that” |  | X |  |
|  | “There was a therapy that was recommended to me maybe at the start of the year, it was called heel drops or painful heel drops? They're on the edge of the steps and you basically flex, you basically flex down and flex up ... Was it 10 minutes a night every night for 12 weeks but in my head that was crazy.” |  | X |  |
| **Identifying with and self-managing AT** | | | | |
| Beliefs surrounding causation  Participants described numerous causes for their AT  Participants described uncertainty in the causal factors associated with the condition | “It might just be the step up in miles like. Might be the mile. Might have gone too…gone from 40 miles up to 70 miles too fast kinda” |  | X |  |
|  | “Obviously there is some weakness there I suppose. Or some, or is it down to running style or something. Footwear, yeah” |  | X |  |
|  | “I don't know. It's a question people often ask. Definitely people would say to you it's an overuse. And well I'm not doing enough for it to be overuse. That's what wrecks me”. |  | X |  |
| Rest as a management approach  Participants noted that rest was often a treatment decision based on their own belief system, made by the health professional or the patient themselves although there was conflicting opinions about the value of rest  Some participants lacked belief in rest as a management approach and expressed a desire to continue running despite advice to the contrary. | “I probably didn’t rest it enough. That’s the only assumption that I can come up with that I didn’t rest it enough. As in I’ve stopped running altogether in the hope that the rest will allow it to recover. The problem is its back to the whole thing of not knowing is the problem” |  | X |  |
|  | “I've gone to a lot of physios and they tell me it’s an overuse injury but I don't think it is ‘cause I've stopped. People say stop and don't do anything for 6 or 8 weeks but if I did stop and went back even for a jog a couple of miles after not doing anything for a period of time id still be in pain like it never stopped” |  | X |  |
| **Looking to the future** | | | | |
| Fear of future prognosis  Participants noted that they were fearful of doing further damage (e.g. tear) if they continued to exercise and or failed to complete their exercise program | “Yeah it could like. It probably ... I'd be thinking that like. By running on it as it is, I think it would. It might get to the stage where it will rupture ... If I don't get it fixed like”. |  | X |  |
| Desire to run  Some participants expressed a desire to return to running at any level | “I suppose being able to get back to running in some shape or form. In an ideal world obviously back to full health and going back to running marathons again. But at the other end of the scale being able to run in some shape or form, any distance just to get out and something”. |  | X |  |

Extraction of findings complete: Yes  No
