# Supplementary material for: Psychological factors associated with foot and ankle pain: a mixed methods systematic review
Source: J Foot Ankle Res. 2022 Feb 3;15:10. doi: 10.1186/s13047-021-00506-3 (PMC8812226; doi:10.1186/s13047-021-00506-3)
Supplement: Supplementary file 3 — Additional file 3:. Supplementary file 2 – List of excluded studies. [file 13047_2021_506_MOESM3_ESM.docx]

Supplementary file 2 – List of excluded studies

| **Author** | **Year** | **Title** | **Reason for exclusion** |
| --- | --- | --- | --- |
| Abhishek | 2010 | Are hallux valgus and big toe pain associated with impaired quality of life? A cross-sectional study | No psychological factors |
| Amin | 2014 | Relationship between psychosocial risk factors and work-related musculoskeletal disorders among public hospital nurses in Malaysia | No psychological factors |
| Anaforotlu | 2014 | How does hallux valgus deformity affect foot function, quality of life and foot pressure? [Turkish, English] | Not English |
| Anaforotlu | 2014 | Relationship between foot pain and quality of life. [Turkish, English] | Not English |
| Arnold | 2011 | Functional Ankle Instability and Health-Related Quality of Life | No psychological factors |
| Awale | 2014 | Severity of foot pain is linked to the prevalence of depressive symptoms: The Framingham foot study | Duplicate |
| Cahalan | 2017 | Biopsychosocial Factors Associated with Foot and Ankle Pain and Injury in Irish Dance: A Prospective Study | pain in other regions |
| Vásquez | 2019 | Influence of psychosocial factors on the experience of musculoskeletal pain: A literature review | Review |
| Dorner | 2018 | How are socio-demographic and psycho-social factors associated with the prevalence and chronicity of severe pain in 14 different body sites? A cross-sectional population-based survey | pain in other regions |
| Fukano | 2020 | Fear Avoidance Beliefs in College Athletes with a History of Ankle Sprain | pain in other regions |
| Gill | 2016 | Predictors of foot pain in the community: the North West Adelaide health study | Pain in other regions. |
| Golightly | 2011 | Association of foot symptoms with self-reported and performance-based measures of physical function: The Johnston County osteoarthritis project | pain in other regions |
| Gunnarsson | 2018 | Clinical pain, abstraction, and self-control: Being in pain makes it harder to see the forest for the trees and is associated with lower self-control | pain in other regions |
| Haouès | 2019 | Severity of hallux valgus and quality of life of elderly people: a descriptive correlational study | No psychological factors |
| Hendry | 2018 | Foot pain and foot health in an educated population of adults: results from the Glasgow Caledonian University Alumni Foot Health Survey | No psychological factors |
| Hill | 2008 | Prevalence and correlates of foot pain in a population-based study: the North West Adelaide health study | Pain in other regions |
| Hiller | 2012 | Prevalence and impact of chronic musculoskeletal ankle disorders in the community | No psychological factors |
| Ho | 2016 | Preoperative PROMIS Scores Predict Postoperative Success in Foot and Ankle Patients | surgery-related |
| Hefland |  | Foot Pain in Later Life: Some Psychosocial Correlates | Review |
| Houston | 2018 | College Athletes With Ankle Sprain History Exhibit Greater Fear-Avoidance Beliefs | pain in other regions |
| Houston | 2014 | Health-Related Quality of Life in Individuals With Chronic Ankle Instability | No psychological factors |
| Irving | 2008 | Impact of chronic plantar heel pain on health-related quality of life | No psychological factors |
| Lazarides | 2005 | Association amongst angular deformities in Hallux Valgus and impact of the deformity in health-related quality of life | No psychological factors |
| Lopez-Lopez | 2016 | Quality of Life Impact Related to Foot Health in a Sample of Older People with Hallux Valgus | No psychological factors |
| Lopez-Lopez | 2015 | Quality of life impact related to foot health in a sample of sea workers | No psychological factors |
| Lopez Lopez | 2016 | Impact on quality of life related to foot health in a sample of menopausal women: a case-control observational study | No psychological factors |
| Lopez-Lopez | 2018 | Evaluation of foot health related quality of life in individuals with foot problems by gender: a cross-sectional comparative analysis study | No psychological factors |
| Mallows | 2017 | Association of psychological variables and outcome in tendinopathy: a systematic review | Review |
| Mallows | 2020 | The association of working alliance, outcome expectation, adherence and self-efficacy with clinical outcomes for Achilles tendinopathy: A feasibility cohort study (the MAP study) | No psychological factors |
| Mao | 2017 | Challenges of managing chronic pain: Start by ensuring realistic expectations | Editorial |
| McCann | 2016 | Resilience and self-efficacy: A theory-based model of chronic ankle instability | Review |
| McCrum | 2019 | An unrecognised masquerader: a retrospective review of people presenting to musculoskeletal physiotherapy with undiagnosed spondyloarthritis | systematic pathology |
| Menz | 2006 | Foot pain in community-dwelling older people: an evaluation of the Manchester Foot Pain and Disability Index | systematic pathology |
| Menz | 2013 | Foot Pain and Mobility Limitations in Older Adults: The Framingham Foot Study | No psychological factors |
| Menz | 2010 | Impact of hallux valgus severity on general and foot-specific health-related quality of life | No psychological factors |
| Mickle | 2011 | Cross-sectional analysis of foot function, functional ability, and health-related quality of life in older people with disabling foot pain | No psychological factors |
| Nakagawa | 2017 | Association of Anxiety and Depression With Pain and Quality of Life in Patients With Chronic Foot and Ankle Diseases | Pain in other regions |
| Owens | 2013 | Risk Factors for Lower Extremity Tendinopathies in Military Personnel | systematic pathology |
| Patterson | 2015 | Concurrent foot pain is common in people with knee osteoarthritis and impacts health and functional status: data from the Osteoarthritis Initiative | Systemic pathology |
| Pearson | 1982 | Health and mental health in older adults | No association between psych and foot pain or foot function |
| Palomo-Lopez | 2017 | Impact of Hallux Valgus related of quality of life in Women | No psychological factors |
| Ross | 2018 | Self-reported social and activity restrictions accompany local impairments in posterior tibial tendon dysfunction: a systematic review | Review |
| Ross | 2019 | Hip extension deficits and psychosocial features in tibialis posterior tendinopathy: a cross sectional study | Abstract |
| Sman | 2014 | Prognosis of Ankle Syndesmosis Injury | No association between psych and foot pain or foot function |
| Sutherland | 2019 | Relationship of Duration of Wait for Surgery and Postoperative Patient-Reported Outcomes for Hallux Valgus Surgery | No association between psych and foot pain or foot function |
| Sutton | 2008 | Association of pain related beliefs with disability and pain in patients with foot and/or ankle pain: a case series | No association between psych and foot pain or foot function |
| Taspinar | 2017 | Comparing the efficacy of exercise, internal and external shoe modification in pes planus: A clinical and pedobarographic study | No psychological factors |
| Walsh | 2016 | Association of Fat Mass and Adipokines With Foot Pain in a Community Cohort | Systemic pathology |
| Walsh | 2017 | Foot pain severity is associated with the ratio of visceral to subcutaneous fat mass, fat-mass index and depression in women | Pain in other regions |
| Yildiz | 2018 | Fear of movement in patients with plantar fasciitis | Abstract |
| Yildiz | 2019 | An investigation of the relationship between pain, foot posture, movement fear, and functional status of the lower extremity with symptom duration in women with ankle inversion injury: Pilot study | Abstract |
